# Price-Denomination Effect: Choosing to Pay With Denominations That Are the Same as the Product Prices

**DOI:** 10.3389/fpsyg.2020.552888

**Published:** 2020-09-09

**Authors:** Elena Reutskaja, Jeremiah Iyamabo, Priya Raghubir, Iñigo Gallo

**Affiliations:** ^1^Marketing Department, IESE Business School, Barcelona, Spain; ^2^Marketing Department, Leonard N. Stern School of Business, New York University, New York, NY, United States

**Keywords:** subjective value of money, denomination choice, judgment anchoring, behavioral economics, field and lab experiments, price

## Abstract

Building on past research on judgment anchoring, we investigate the effect of price information on consumers’ choice of denomination when making a purchase. Across seven experiments, including two in the field (*N* = 4,020), we find that people tend to purchase with denominations that are the same as the product prices. They use larger denominations for higher priced products that are priced at the value of the denomination held, and smaller denominations for lower priced products that are priced at the value of the smaller denomination held. The effect is not explained by storage or purchase convenience. We propose the “price-denomination effect” is driven by consumers anchoring on product price and then choosing the denomination that matches the anchor. The effect replicates across participants from different continents (United States, Europe, and Africa) and samples (online panelists, and actual consumers), as well as prices in different currencies (United States $, €, and Nigerian Naira). We further demonstrate that people’s preference for denominations also affects the choice of the form of payment used: cash versus card. Consumers are more likely to use cash (vs. card) when product price is exactly the same as a denomination held. We conclude with a discussion of theoretical and practical implications.

## Introduction

Recently, ATMs in the U.S and Europe have started allowing customers a choice of denominations when they withdraw money from their bank accounts; an option that was earlier only available at the teller. For example, when withdrawing $200, the customer has the option to withdraw ten $20 bills or four $50 bills. Can the type of denomination chosen affect how the money will be spent? And is the choice of denomination used to spend a function of the price of the products that customers intend to purchase?

The use of cash in the marketplace is an interesting phenomenon (Prelec and Simester, 2001; Raghubir and Srivastava, 2002; Amromin and Chakravorti, 2009). Despite the growing availability of new payment methods, and despite the arguments from some economists and policymakers for the phasing out of paper money (Rogoff, 2016), cash remains the most heavily used retail payment instrument (Matheny et al., 2016). In developed economies such as the USA, Japan, and Singapore, cash remains the dominant mode of payment, with around 85% of payments made with cash globally (Wheatley, 2017). Because cash continues to be relevant to people, understanding how people make decisions regarding the use of cash is a relevant pursuit.

It is particularly relevant given that there is a misalignment between the predictions of traditional economic model assumptions and people’s actual behavior. On the one hand, according to standard economics, money is money, irrespective of payment method, currency, or denomination. On the other hand, research on the subjective value of money consistently shows otherwise. For instance, money is judged beyond its actual denomination value: people assign greater value to money based on physical properties, such as the size of coins (Bruce et al., 1983) and spend more when the face value of a foreign currency is a fraction of one’s home currency (Raghubir and Srivastava, 2002). Additionally, people’s perception of the economic value of bills is primarily affective, rather than numerical (Giuliani et al., 2018). Consumers also estimate higher purchasing power due to money familiarity (Alter and Oppenheimer, 2008), are more likely to spend, and spend more, when they have smaller rather than larger denominations (Mishra et al., 2006; Raghubir and Srivastava, 2009), and spend less when paying with cash versus credit cards (Prelec and Simester, 2001; Raghubir and Srivastava, 2008).

The research presented here falls within this program on the subjective value of money. This paper examines how people choose different denominations of cash: a new effect termed the “price-denomination effect.” We propose that the decision of which denomination to use will be a function of the price of the product. Specific denominations will be more likely to be used to purchase a product when their value is equal to its price. For example, consumers will use a larger denomination to buy a product, which has the same price as the value of the denomination, even when smaller denominations held are inconvenient to store.

We examine how price information affects people’s choice of denomination to pay for a purchase (e.g., with one €50 bill or five €10 bills) and choice of form of payment (cash vs debit card). Seven experiments (including two field experiments, *N* = 4,020), test our predictions using participants from different continents (United States, Europe, and Africa) and samples (online panelists, and Nigerian consumers), as well as prices in different currencies (United States $, €, and Nigerian Naira). We propose that once consumers have decided to purchase, they use price information as a judgment anchor and this tendency to anchor on price information influences their choice of denomination. For instance, if a consumer has five $10 bills and a $50 bill and has decided to purchase a $10 product, she is more likely to pay using the $10 bill; but if she has decided to purchase a $50 product, then she is more likely to pay using the $50 bill, violating the descriptive invariance of money principle. We test this proposition in study 1.

We further propose that anchoring on price information is one of the drivers of the effect and elicits faster responses when choosing the denomination with which to purchase, indicating that when there is a match between the price and the denomination carried, the decision of which denomination to use is faster to make (study 2). We demonstrate that having the price be exactly the same as the denomination carried is not a necessary condition for the effect to occur and that denominations should be “close enough” to the price anchor for the effect to hold (the effect holds for prices which are 10 – 20% below the denomination value, study 3). We further show that in spite of the fact that most people judge smaller denominations as easier to purchase with, the majority of participants decide to pay with larger, not smaller, denominations. Therefore, purchasing (transactional) convenience is unlikely to explain the results. We go on to further show that storage (carrying) convenience is also an unlikely mechanism behind the effect (study 4). Overall, the price-denomination effect holds even when controlling for purchasing and carrying convenience.

One alternative explanation for our effect may be value-matching. That is, people would use value as a heuristic to make a purchase. Both larger denominations and more expensive purchases might be seen as more valuable to the consumer, and, therefore, matching value of the product and value of denomination might drive our effect. Study 5 finds that matching of denomination value and value of the product to be purchased (e.g., a Valentine gift or a gift for a jerk boss) are unlikely to explain the effect. Finally, we suggest that the price-denomination effect could extend to the choice of form of payment (cash vs card). We demonstrate that consumers’ payment preference (cash versus card) shifts depending on whether the price they encounter is the same as the denominations they hold. Consumers are more likely to pay with cash over card when the denomination they hold is exactly the same as the price of the product to be purchased, and they are more likely to use a card for their purchase when the price and denomination at hand are not the same. We note that our theory does not make predictions for all possible scenarios and mixes of prices and denominations that consumers can encounter. Our theory only makes clear predictions for situations in which a consumer wants to buy a product, the price of which is exactly the same as one of the denominations she holds. To examine the boundary conditions of our effects, we also include an examination of the price-denomination effect where we vary the price of the purchase by lowering it 10, 20, and 30% and, therefore, distance it from the denomination carried. We find that our effect holds also for prices that are up to 10% below the denomination at hand. In other situations, not examined in this paper, consumers might either be indifferent between the use of two denominations at hand, supporting the money invariance principle, or be more likely to spend smaller denominations, supporting the “denomination effect”, which we discuss below.

Our work contributes to the research on the subjective value of money by demonstrating violations of the descriptive invariance of money in the domain of which denomination to pay with. We also contribute to the price anchoring literature by documenting a new downstream consequence of prices on consumers’ behavior. Finally, our theory and supporting evidence are in line with the mental accounting perspective (Thaler, 1985; Prelec and Simester, 2001): consumers will use small denominations when prices are equal to the small denominations at hand, and large denominations for large purchases priced the same as the larger denomination. Our findings specifically add to the literature on the “denomination effect” (Raghubir and Srivastava, 2009) which suggested that one of the reasons people were more likely to make a purchase when they held smaller denominations was because they wanted to exert self-control and did not want to lose track of how much money they had (see also Raghubir et al., 2017). Other reasons proposed for the same effect include perceptual fluency (Mishra et al., 2006), and feelings of smaller notes being “dirty” as they are in greater circulation than larger notes (Di Muro and Noseworthy, 2013). The vast majority of the studies in previous investigations of the denomination effect had choice sets where the value of products was closer to the smaller denomination that participants had been given. It is plausible that the prices of the majority of products used in previous studies were close to the smaller denominations which led to an increased likelihood of spending in the lower (vs. higher) denomination conditions. As such, the match between price and denomination may be an additional factor explaining the denomination effect.

Knowing how the public uses different denominations also has important practical implications. For example, knowing how denominations are used is important for central banks who decide on money issuing and maintenance policies. In addition, banks can use this information to decide which denomination to stock at both the bank tellers as well as at ATMs. For example, at an upscale mall, retailers might benefit if ATMs issued larger denominations, while outdoor retail markets like food markets and farmers markets may benefit if ATMs closer to them issued smaller denominations. These results can also help managers decide on pricing policies for individual products and bundled options. For example, in countries where the commonly available denominations are 20, 50, and 100, managers may wish to create bundled or unbundled options that are closer to 50 or 100 than to 75. In the following sections, we first discuss past research on judgment anchoring. The studies are then described. We conclude with a discussion of the results, theoretical contributions, managerial implications, and opportunities for further research.

## Theoretical Framework and Hypotheses

Judgment anchoring has been defined as an effect that occurs when individuals are biased toward an arbitrary value before making a numerical estimate (Jacowitz and Kahneman, 1995). The selective accessibility model proposed by Strack and Mussweiler (1997) argues that anchoring occurs when an anchor activates a target response, with anchors being illustrations of semantic priming. Priming refers to situations where information that is activated becomes more accessible when solving tasks compared to non-activated information. Applying their model to point-of-purchase decisions, we suggest that the prices consumers encounter can function as judgment anchors and affect subsequent judgments (cf. Mussweiler and Strack, 1999; Lin and Chen, 2017; Köcher et al., 2019).

While making a judgment, people resort to readily accessible information (e.g., the anchor), which carries through to their decision. Accordingly, responses tend to converge toward the anchor. Anchors increase the saliency of information irrespective of whether the anchor is arbitrary (Ariely et al., 2003), encountered in the environment (Jacowitz and Kahneman, 1995) or self-generated (Epley and Gilovich, 2005). Research in the consumer domain has shown that consumers anchor on price information when making purchase decisions (e.g., Morwitz et al., 1998). Anchoring effects involving price information have also been observed in bidding contexts. Consumers spend more and recall lower costs when exposed to a surcharge compared to control conditions (Morwitz et al., 1998; Chakravarti et al., 2002). More recently, Jung et al. (2016) demonstrated that when consumers anchored on higher bonus prices, they spent more on average than when they anchored on lower bonus prices. In addition, the proportion of consumers’ spend allotted to charity versus the retailer was influenced by whether they encountered a higher or lower anchor. Price anchoring is just one of the many ways in which prices influence consumers’ decisions or in which consumers use prices. Consumers use price cues to estimate product quality (Zeithaml, 1982). They also engage in price search in an attempt to increase savings (Stigler, 1961). Further, consumers’ tendency to disregard prices with −99 endings leads them to underestimate the actual prices of products; thereby overspending when they are offered clearance sales (Schindler and Kibarian, 1996). Other research from Thomas and Morwitz (2005) shows that consumers misattribute the magnitude of price discounts to the ease of computing the difference between a regular price and a sale price. In a different but related context, Choi et al. (2019) found that numeric information with −99 endings increased consumers’ unhealthy food consumption compared to numeric information with −00 endings. It, therefore, goes to reason that prices exert other forms of influence on consumers at a point of purchase.

Of particular concern to the present research, is the choice of which denomination to purchase with when consumers have decided to pay for a product or service. Relating the research on judgment anchors to the price contexts involving choice of denominations, we propose that, when making a purchase, price acts as a factor that is contextually available and affects choice of denomination. We argue that the prices consumers encounter represent salient environmental anchors both because such prices are readily available (Tversky and Kahneman, 1974) and because they are relevant (Davis et al., 1986). We suggest that because price information is salient in shopping contexts (Thomas and Morwitz, 2005), it activates the saliency of denominations consumers possess, provided prices and denominations match each other (e.g., price of $50 and denomination of $50). Given that people tend to rely on information that is activated in memory (Sedikides and Skowronski, 1991), they should also exhibit a tendency to purchase with the denominations the person possesses, which are activated by the price. This implies that if denominations are not the same as the price, consumers will be less likely to rely on price information in choosing the denomination with which to purchase. To put it more formally:

H1: Consumers are more likely to purchase with the denomination that is a match with the price of the product when selecting between two denominations at hand.

By “match” we mean the price that is exactly the same as the denomination at hand. We further use the term “match” and the expression “price is exactly the same as the denomination” interchangeably.

Epley and Gilovich (2001) demonstrated that people respond faster the more they rely on anchors that readily come to mind. In contrast, they respond slower when their final responses deviate from readily available anchors. Thus, in line with existing theory, we further hypothesize:

H2: People decide on which denomination to choose faster when prices are a match with the denomination they possess, compared to when they are not.

## Empirical Overview

We conducted seven studies to test the price-denomination effect and the role of price as an anchor in the choice of denomination (we also report three additional studies in the Supplementary Appendix replicating the studies in the main manuscript under slightly different conditions and contexts). In field studies 1A-1B, we test H1 using actual consumers in Africa where we could take advantage of the lower cost of living, and use relatively large denominations in an economically feasible manner. Study 2 tests our proposed mechanism – judgment anchoring. Studies 3-4 test whether results can be extended beyond exact matching (study 3) and storage convenience (study 4). Study 5 rules out that the transfer of ownership value from denominations to the product moderates our proposed effect. Finally, study 6 extends the price-denomination effect to choice of payment form: cash vs. card. Table 1 shows a summary of all the study descriptions. Data, study protocols and analysis codes are publicly available at https://osf.io/syvrm/(the flows of the field studies are described in detail in the text directly).

**TABLE 1:** Study Descriptions (1 – 6).

Study	Denominations	Spend Level	Products	Prices	*N*	Results
1A	N50 & 5 × N10	N100	One pen	N10	399	Results^1^: 98%
			A set of five pens	N50		17%

1B	N200 & 4 × N50	N400	phone voucher	N200	388	2%
			A set of five pens	N50		100%

2	1 × $50 & 5 × $10	$100	Shampoo	Exact Match: $10	1,204	Results^2^: 10.04 secs
				Equidistant: $30		11.03 secs
				No price		10.61 secs
			Perfume	Exact Match: $50		7.03 secs
				Equidistant: $30		9.35 secs
				No price		9.87 secs

3	$100 & 5 × $20	$200	Compact Camera	$100, $90, $80, $70	881	Results^3^: 70%, 83%, 60%, 56%,
	$50 & 5 × $10	$100	Perfume	$50, $45, $40, $30		88%, 87%, 55%, 51%

4	$50 & 50 × $1	$100	Perfume	$50	550	Results^1^: 29%
			Shampoo	$10		85%
	$100 & 100 × $1	$200	Camera	$100		32%
			Shampoo	$10		93%

5	5 × €10 & 1 × €50	€100	Gift: Jerk boss	€10	438	Results:^1^ 90%
			Gift: Valentine	€10		91%
			Gift: Jerk boss	€50		24%
			Gift: Valentine	€50		19%

6	10 × $10	$100	Taxi ride	$10	160	Results^4^: 76%
	10 × $10			$50		40%
	2 × $50			$10		46%
	2 × $50			$50		63%

*^1^Proportion paying with the smaller denomination, ^2^Participants’ reaction time when choosing which denomination to purchase with, ^3^Proportion paying with the larger denomination, ^4^Proportion paying with cash.*

The method of analysis across all studies is to examine differences in the percentage of participants using a given denomination across price conditions. Additionally, we examined whether the percentage of participants who chose the non-matching denomination varied significantly from 50% (given there was a choice of two denominations, 50% represents a random choice or guessing). In no study was there any evidence for over 50% of participants purchasing with the non-matching bill (see Table 1).

A meta-analysis testing mean p-values using Rosenthal’s (1978) approach shows that the price-denomination effect is significant [*z* = 8.78, if we use *N* = 26; or *z* = 5.17, if we use *N* = 9, where *N* = number of studies used for analysis, *ps* < 0.001 for both, see Supplementary Appendix 8 for details.]

## Study 1. Establishing the Effect With Actual Purchases

The objective of studies 1A-1B was to test the price-denomination hypothesis (H1) in actual retail settings using relatively large sums of money for the participant population. We took advantage of the cost of living in Nigeria and conducted our studies in Lagos. We used the local currency (naira, denoted as “N”) with an exchange rate of $1: N360.68. By conducting the study in Nigeria we were able to use large denominations in a real life setting (e.g., participants handled a 200N bill which is the 3^*rd*^ largest denomination in the country) – something that would not be possible to accomplish in a Western European country or North America due to budget constraints. Study 1A uses lower denominations and price levels than study 1B. In line with our theoretical framework, we expect consumers to choose the denomination that matches product price.

### Study 1A: Method

#### Participants and Design

Three hundred and ninety-nine students and workers (female = 51%) who were responding to an on-campus sales promotion at two Lagos universities were assigned at random to a two-cell (price: N10 vs. N50) between-subjects design.

#### Materials and Procedure

Upon arrival, participants received N100 (≈ $0.28/ c or €0.23/ c) in cash in an unsealed brown envelope: one N50 bill and five N10 bills. They were told they must purchase one product and could select one of two products available at the promotion, depending on the price condition they were assigned to, using their money and could leave with the rest of their money. In the price = N10 condition, participants saw three pens (blue, black and red), and had to purchase one of these pens. In the price = N50 condition, they had to purchase one of two sets of five pens (set 1: two black, two blue and one red pen versus set 2: four red and 1 black pen). Finally, each participant was issued a receipt and thanked. Assistants, blind to the study’s objective, manually recorded the choice of denomination (N10 or N50) used to pay for the N10 or N50 purchase using the duplicate copy of the receipt. Four observations were excluded from the analyses due to errors in recording participants’ responses leading to a usable sample of *N* = 395.

### Results

**FIGURE 1:**
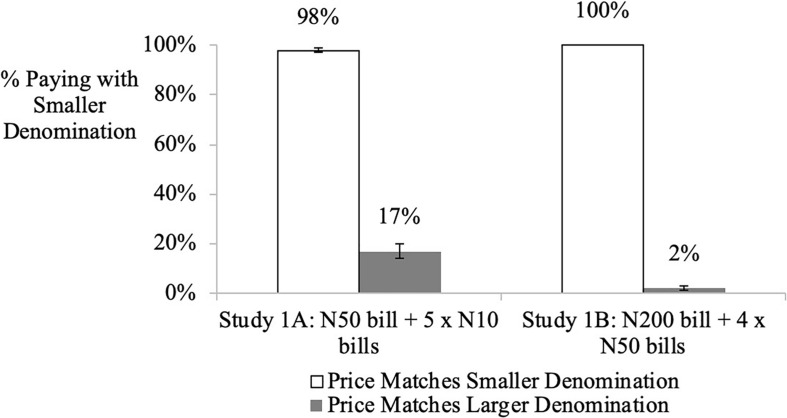
Influence of “price-denomination effect” on denomination choice (studies 1a and 1b). Error bars denominate std. errors. The results were similar for studies 1A and 1B. For instance, in study 1A, when the price was N10, 98% of consumers chose to purchase with the matching N10 bill they possessed. However, when the price was N50, the proportion of consumers purchasing with the same N10 bills decreased to 17%.

The proportion of participants purchasing with the smaller N10 bill was 98% (versus 50%; *z* = 13.46, *p* < 0.001) in the N10 price condition versus 17% in the N50 price condition (versus 50%; χ^2^(1) = 263.34, *z* = −9.38, *p*s < 0.001), see Figure 1. This provides initial support for H1.

### Discussion

Participants chose the denomination that matched the price of the product they had to purchase, consistent with H1. Study 1B examines the generalizability of the effect using a higher spending level, a higher set of prices, and larger denominations.

### Study 1B: Method

#### Participants and Design

The design was identical to study 1A, with the exception of the denominations and prices, which were higher. Three hundred and eighty-eight students and workers participated in an on-campus sales promotion. Participants received N400 in a single N200 bill and four N50 bills. Participants were assigned, at random, to one of two price conditions in a between-subjects design. In the price = N50 condition, participants chose between two sets of pens, similar to study 1A. In the price = N200 condition, participants chose a telephone voucher from one of the four existing telecoms providers. We could not use the same product in different price conditions as prices are different and there was no equivalent product available at both price levels.

### Results

In the price = N50 condition, 100% of participants purchased with the smaller N50 bill, as compared to only 2% (versus 50%) in the price = N200 condition (χ2(1) = 185.25, *z* = −13.68, *p*s < 0.001, significantly less than 50%), see Figure 1.

### Discussion

Study 1B replicated the effect of study 1A with high spending levels and higher prices. Overall, study 1 demonstrated in the field that people are likely to use the denomination that is the same as the price of the product they need to purchase. This is consistent with H1. We note that the number of options varied between conditions as it was not feasible to use the same number of options: we used 3 single pens vs 2 sets of pens, or we used all the colors of the pens available (3) and all the vouchers available in the store (4). We could not limit the number of vouchers to three to match the number of pens because, otherwise, some of the participants would be at a disadvantage, as they would not have the option of the phone voucher of their network. We, however, did not expect that this difference in number of options would create a systematic bias, but we recognize this as a limitation of this study. Later studies use an identical number of choice options. Supplementary Appendix 1 reports an additional study testifying to the generalizability of this effect using samples from a Prolific Academic panel, the euro currency, a wide variety of products, and constant number of options across conditions.

One of the limitations of the field study is that we were unable to elicit the mechanism(s) driving the price-denomination effect. In studies 2-5 we explore possible processes underlying the effect. To begin, Study 2 examines price anchoring as a potential mechanism. It explores whether the effect persists when the price anchor is removed and whether consumers are faster at choosing denominations that match the prices encountered. We also include controls for product familiarity and attitude, as these can change the extent to which people think intuitively (Zajonc and Markus, 1982).

## Study 2. Judgment Anchoring

The price-denomination effect proposes that prices that are the same as the denominations at hand are relevant contextual cues that will affect the choice of denomination to use. Previous research suggests that people respond faster to readily available anchors (Epley and Gilovich, 2001). Accordingly, in this study we explore whether consumers choose between denominations at hand similarly when a price anchor is available (vs not available) and whether consumers make faster decisions when there is a closer match between prices and denominations.

### Method

#### Participants and Design

One thousand two hundred and four Amazon Mechanical Turk participants (female = 49%, *M*_*age*_ = 38.02, *SD* = 12.95) were recruited to participate in this study in exchange for modest compensation. They were assigned at random to one of six conditions using a between-subjects design that manipulated the price levels of two products (shampoo vs. perfume) and the type of price information (exact match vs. equidistant vs. no price).

#### Materials and Procedure

Participants read instructions asking them to imagine they wanted to purchase either a luxury shampoo or a perfume. Participants were further asked to imagine they held $100 as a $50 bill and five $10 bills, and were shown images of the bills they had. Depending on condition, participants saw one of the three price anchors. In the shampoo conditions the price levels were: $10 (exact match anchor), $30 (equidistant anchor) or no price. In the perfume conditions, the prices were $50 (exact match anchor), $30 (equidistant anchor) or no price. Though the “no price condition” is not realistic as consumers usually know how much each product costs, from a theoretical point of view, this condition allows for a test of whether there is a main effect of product and whether people have approximate price anchors in their minds. Participants then indicated their choice of denomination. We measured their response latencies (in seconds). Following this, participants responded to product attitudes (“How much do you like ___?”) adapted from Irmak et al. (2010) and product familiarity (“How often do you use ___?”) adapted from Teixeira et al. (2014) measures, both measured on 100-point slider scales (0 = “not at all”/100 = “very much”). Finally, participants indicated their age, gender, and income level. Fifty-nine respondents (5% of participants) failed an attention check and were excluded from the analyses, leaving a usable sample of *N* = 1145.

### Results

#### Denomination Choice

**FIGURE 2:**
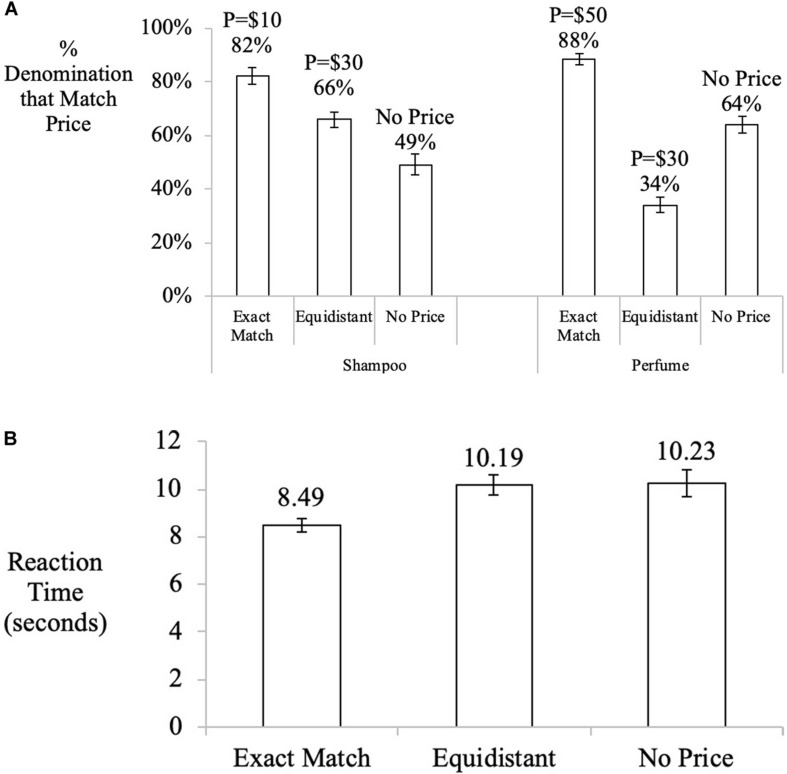
**(A)** Study 2. Influence of price-denomination effect on denomination choice for six conditions that manipulate price levels. Error bars denominate std. errors. All participants were endowed with a $50 bill and five $10 bills. The *y*-axis represents the proportion of participants purchasing with denominations that “matched” different product conditions (that is, matched $10 in shampoo condition, and $50 in perfume condition). The *x*-axis represents the type of price information encountered and the product conditions participants were assigned to. **(B)** Study 2. Evidence for judgment anchoring mechanism on price-denomination effect using response latencies on purchase decisions as the dependent variable (in seconds). Error bars denominate std. errors.

The dependent variable was the proportion of participants who purchased with the denomination that was equal to the product price (coded “1”; otherwise “0”). As shown in Figure 2A, the proportion of participants who purchased with the denomination when the denominations matched price ($10 for shampoo, $50 for perfume) was significantly higher (85%) than those purchasing with $10 bills and $50 bills respectively, when the price was $30, or equidistant from the two denominations (50%, χ2(1) = 109.15, *p* < 0.001), as well as those in the no price anchor condition (57%, χ2(1) = 76.06, *p* < 0.001). There was a marginally significant difference between the no price and price $30 conditions (50% vs. 57%, χ2(1) = 3.44, *p* = 0.063). In the no price anchor condition, respondents were indifferent in their choice of denominations when purchasing shampoo (49%, χ2(1) = 0.048, *z* = −0.22, *p* = 0.413), but were more likely to use their $50 bill to purchase the perfume (64%, χ2(1) = 15.67, *z* = 3.96, *p* < 0.001; overall 49% vs. 64%: χ2(1) = 8.77, *p* = 0.003). We also conducted logistic regressions estimating denomination choice as a function of price anchors controlling for covariates. The results indicate that our findings are robust. Participants were more likely to use larger denomination for purchasing high priced product (log-odds = 1.50, *p* < 0.001, Supplementary Table A4) and less likely to use larger denomination for purchasing a low priced product (log-odds = −1.56, *p* < 0.001, Supplementary Table A5) in comparison to the no-price condition when an exact price anchor was available. For both high- and low- priced products, people were less likely to use the larger denominations when an equidistant price anchor was available in comparison to the no-price condition (log-odds = −1.23, *p* < 0.001 for high-priced product; and = −0.70, *p* = 0.001, for the low-priced product, Supplementary Tables A4, A5). We found no significant effect of the covariates of familiarity and attitude. We also did not find any significant effects for gender or income. The effect of age was significant, but small, for the higher-priced product (log-odds = 0.02, *p* = 0.038, see Supplementary Appendix 2, Supplementary Tables A3–A5).

#### Response Latencies

Participants’ response latencies (in seconds) per condition are presented in Figure 2B. We performed an analysis on the log transformation of the response latencies as the response latencies were skewed. The results are robust when the ANOVA analysis is done on the non-transformed response latencies (see Supplementary Appendix A2). A one-way ANOVA on a log transformation of reaction time as a function of price information including all covariates, revealed a main effect of price information (*F*(2, 1137) = 8.10, *p* < 0.001, η^2^ = 0.01), product attitude (*F*(1, 1137) = 4.05, *p* = 0.044, η^2^ = 0.00), product familiarity (*F*(1, 1137) = 8.07, *p* = 0.005, η^2^ = 0.01), age (*F*(1, 1137) = 102.99, *p* < 0.001, η^2^ = 0.09), gender (*F*(1, 1137) = 5.24, *p* = 0.022, η^2^ = 0.00), and income (*F*(1, 1137) = 7.64, *p* = 0.006, η^2^ = 0.01), indicating that the speed at which participants responded to the product varied depending on whether prices matched denominations held. The effect of matching was similar when the covariates were excluded from the model (*F*(2, 1142) = 7.63, *p* = 0.001, η^2^ = 0.01).

On average, participants who faced a price that was an exact match for the denomination they carried responded faster (*M* = 1.95) compared to those who saw the $30 price (*M* = 2.11, *t*(763) = −3.57, *p* < 0.001, Cohen’s *d* = 0.26, *t*-test of log-transformed time in seconds), or those who saw no price (*M* = 2.10, *t*(762) = −3.23, *p* = 0.001, Cohen’s *d* = 0.23).

We also conducted regressions estimating the log of response time as a function of price anchors controlling for covariates. The results indicate that our findings are robust and that participants in the exact match condition responded faster than in the non-price condition (log-odds = −0.30, *p* < 0.001), see Supplementary Appendix A2, Supplementary Table A6. The regression analysis also indicates that this difference is mainly driven by the high-priced product (log-odds of interaction of low-priced Shampoo_Product^∗^Exact_Match_Anchor = 0.34, *p* < 0.001). There is also a significant, though small, positive effect of attitude (log-odds = 0.002; *p* = 0.026), and age (log-odds = 0.01; *p* < 0.001) and a small negative effect of income (log-odds = −0.01, *p* = 0.008).

### Discussion

Study 2 results show that people choose denominations that match the price of purchases being considered more often than denominations that do not match those prices, and make these decisions faster. These results are consistent with the idea that prices serve as judgment anchors. An interesting result was when no price was present: consumers were equally likely to choose either denomination in the case of the shampoo but they were more likely to use their $50 bill for the perfume. This suggests the possibility that people may have approximate price anchors in their minds, with some products having more salient price anchors than the others. For example, participants might believe that perfume is more likely to be priced around $50. However, it might be harder for people to rapidly assess the price of a luxury shampoo as the prices of luxury shampoos might vary. Without a clear price anchor in mind, participants might simply use either denomination at random to make a purchase. Exploring the role of implicit anchors may be an interesting avenue for future research. A reasonable criticism of our designs so far is that in typical retail settings prices are not an exact match of the denominations that consumers hold. For example, the .99$ phenomena (see, Schindler and Kirby, 1997; Thomas and Morwitz, 2005, 2009), is wide-spread, as are prices that require a mix of denominations (e.g., $80, $40). Janiszewski and Uy (2008) have also observed that precise price anchors led individuals to overestimate costs of a wide range of products more than rounded anchors. Study 3 investigates the boundary conditions of the effect of price anchors by using prices that are 10, 20, and 30% below the denominations at hand.

## Study 3. Prices Lower Than the Denomination Value

### Method

#### Participants and Design

Eight hundred and eighty-one participants (female = 51%, *M*_*age*_ = 37.54, *SD* = 11.91) were recruited from Amazon Mechanical Turk in exchange for modest compensation. Participants were assigned at random to one of eight conditions in a between-subjects design, where participants encountered one of two possible products (camera versus perfume) and four possible prices.

#### Materials and Procedure

We adopted the same cover story used in study 2. In the camera conditions, participants encountered one of four possible prices (starting at $100 and reducing by $10 [10%] for each condition: $100, $90, $80 and $70). Similarly, in the perfume conditions, participants encountered one of four possible prices (starting at $50 and reducing by $5 [10%] for each condition: $50, $45, $40, $35). Participants were told that they had a hypothetical spending budget of $200 and $100 in the camera and perfume conditions respectively. In the camera conditions, the denominations were a single $100 bill and five $20 bills. In the perfume conditions, participants had a single $50 bill and five $10 bills. We expected participants to be more likely to purchase with the larger denomination held in each price condition.

In addition to the measures on product familiarity and product attitude used in study 2, we aimed to control for purchase and storage convenience. Participants rated both the large and the small bills on purchase convenience (“How convenient is $__ bill for making purchases?”), and on storage convenience (“How convenient is $__ bill for carrying in a wallet?”), both on a continuous scale from 1 (“not very much”) to 10 (“very much”). Finally, participants indicated their age, gender, and income levels.

### Results

#### Denomination Choice

**FIGURE 3:**
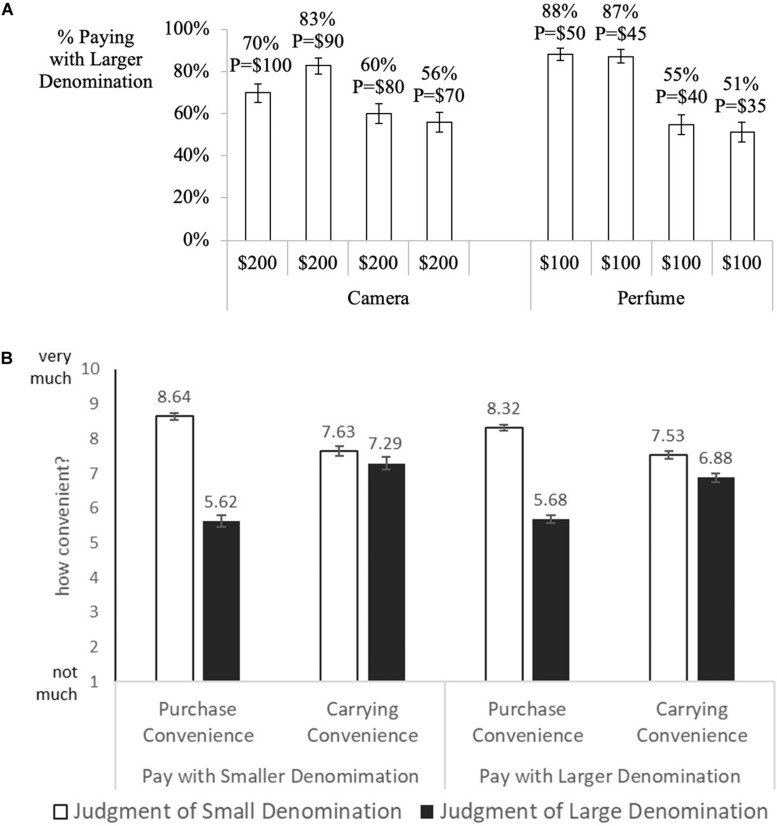
**(A)** Study 3. “Price-denomination effect” when prices fall below 10% - 30% of the denomination value. Error bars denominate std. errors. The *y*-axis represents the proportion of participants purchasing with the larger denomination. The *x*-axis represents the respective amounts participants in the camera and perfume conditions were endowed with. **(B)** Study 3. Judgement of purchase and storage convenience by type of bill one paid with. Error bars denominate std. errors.

The dependent variable was the proportion of participants paying with the larger denomination. As shown in Figure 3A, participants in the camera conditions were more likely to purchase with the larger denomination when the prices were up to 20% below the largest denomination value ($100 camera: 70% [χ2(1) = 17.6, *z* = −4.20, *p* < 0.001]; $90 camera: 83% [χ2(1) = 47.13, *z* = −6.86, *p* < 0.001]; $80 camera: 60% [χ2(1) = 4.77, *z* = −2.18, *p* = 0.015]. However, for the $70 camera, the effect was attenuated to being marginally significant (56%, χ2(1) = 1.75, *z* = −1.32, *p* = 0.093).

Participants in the perfume conditions were more likely to purchase with the larger denominations but only when the price was reduced by ten percent of the larger denomination value ($50 perfume: 88% [χ2(1) = 64.15, *z* = 8.01]; $45 perfume: 87% [χ2(1) = 61.13, *z* = 7.82], *p*s < 0.001). However, when prices were further reduced by 20% and 30%, participants were indifferent in their choice of denomination ($40 perfume: 55% [χ2(1) = 1.11, *z* = 1.05, *p* = 0.146]; $35 perfume: 51% [χ2(1) = 0.08, *z* = 0.29], *p* = 0.387). Logistic regressions controlling for the convenience measures as well as other covariates indicate that the effect is robust, suggesting that people facing prices that matched exactly or were 10% below the larger denomination at hand were more likely to use larger denominations than when price was 30% below the denomination value (contrasts P = $100 vs P = $70, log odds = 0.70, *p* = 0.020; P = $90 vs P = $70 log odds = 1.57, *p* < 0.001; P = $50 vs P = 35$ log odds = 1.97; *p* < 0.001; P = $45 vs P = 35$ log odds = 1.98; *p* < 0.001, Supplementary Appendix 3, Supplementary Tables A12, A13). We find that no covariates were consistently significant for both products used in this study. However, some individual covariates were significant for one product but not the other.

In order to understand the influence of convenience, we compare how people judge larger and smaller denominations in terms of their carrying and purchase convenience. On average, people judged smaller denominations to be more convenient to carry (*M*_small__er_____denom_ = 7.56 vs. *M*_large__r_____denom_ = 7.01, *t*(880) = 4.09, *p* < 0.001) and more convenient to purchase with than the larger denominations (*M*_small__er_____denom_ = 8.42 vs. *M*_large__r_____denom_ = 5.66, *t*(880) = 23.48, *p* < 0.001). We further compared the responses from participants who chose to pay with the larger denominations (*n* = 607) to those who chose to pay with the smaller ones (*n* = 274). Participants who chose the smaller denomination rated smaller denominations equally convenient to carry as the larger ones (*M*_small__er_____denom_ = 7.63 vs. *M*_large__r_____*denom*_ = 7.29, *t*(273) = 1.48, *p* = 0.139), and participants who chose the larger denomination rated smaller denomination more convenient to carry (*M*_small__er_____denom_ = 7.53 vs. *M*_large__r_____denom_ = 6.88, *t*(606) = −3.91, *p* < 0.001). Regarding purchase convenience, participants in both groups rated the smaller denomination as more convenient for making purchases than the larger denominations (those who chose the small denomination: *M*_small__er_____denom_ = 8.64 vs. *M*_large__r_____denom_ = 5.62, *t*(273) = −15.66, *p* < 0.001; those who chose the larger denomination: *M*_small__er_____denom_ = 8.32 vs. *M*_large__r_____denom_ = 5.68, *t*(606) = −18.03, *p* < 0.001), see Figure 3B.

### Discussion

This study confirms and builds on the results of the previous two studies. We replicated and extended the price-denomination effect for prices that were 10% below the denomination value. Thus, exact match of denomination and price is not a necessary condition for the “price-denomination effect” to occur. Prices that are not an exact match but are close matches to denominations held may also serve as anchors guiding the choice of denomination to use. Study 3 also provides preliminary evidence that when prices are 20–30% lower than the denomination carried, consumers are indifferent in terms of choosing which denomination to pay with. Said differently, the diagnosticity of prices as anchors reduces if they are not an exact or a close match to the price.

We replicated the results of study 3 in a lab setting using prices ending at .99 and in a field setting in Africa using a simpler design and an actual purchase (for full study details, see Appendices 4 and 5). The studies lend further support that price and denomination do not need to be exactly the same for the effect to hold.

Results on the measure of convenience further indicate that purchase convenience is unlikely to explain the effect reported here. A majority (69%) of respondents choose to pay with larger denominations even though larger denominations were consistently judged as less convenient to purchase with than the smaller bills. On the other hand, results from study 3 on storage convenience are not conclusive. Larger denominations were viewed as equally or less convenient to carry than smaller denominations. Therefore, an unanswered question is whether the price-denomination effect could be explained by consumers’ tendency to manage the amount of cash they have at hand. Thus, in study 4, we manipulate the storage convenience of smaller denominations (and make smaller denominations far less convenient to carry than larger ones) and test whether storage convenience could drive consumers to get rid of several smaller bills at the earliest opportunity (Mishra et al., 2006).

## Study 4. Does Storage Convenience Predict the Effect?

The objective of study 4 was to test whether the “price-denomination effect” is influenced by storage convenience. We stretched the number of smaller bills to an extreme: we examined conditions in which participants had fifty or one hundred $1 bills. Holding so many $1 bills is not realistic; nevertheless, the aim was to experimentally determine the role of storage convenience versus prices that serve as an anchor. We predict that, contrary to the intuition that consumers will get rid of so many smaller bills for the sake of storage convenience, people are more likely to purchase with denominations that serve as anchors and match the prices they encounter.

### Method

#### Participants and Design

Five hundred and fifty participants (female = 45%, *M*_*age*_ = 36.41 years, *SD* = 11.70) were recruited from Mechanical Turk in exchange for modest compensation. Participants were assigned at random to one of four conditions in a 2 (purchase price: price matches the larger denomination vs. not) × 2 (spend level: $100 vs. $200) between-subjects design.

#### Materials and Procedure

The cover story was adapted from study 2. Depending on their assigned condition, participants were asked to imagine they had decided to purchase a product whose price was either the same as the larger denomination carried ($50 perfume in the $100 spend level condition, or $100 camera in the $200 spend level condition), or was $10 (shampoo for both spend level conditions). Participants were told how much money they hypothetically possessed and the denominations in which they had it. As with earlier studies, participants had to decide which denomination they would use, given they could only purchase one unit of the product and had no other means of payment.

Participants in the $100 spend level conditions were shown images of a $50 bill and fifty $1 bills, and indicated which denomination(s) they would use to buy either a perfume (price = $50) or a shampoo (price = $10). In the $200 spending level conditions, participants were provided with a $100 bill and one hundred $1 bills, and decided which denominations to use in purchasing either a camera (price = $100) or a shampoo (price = $10).

In addition to the measures on product familiarity and product attitude used in study 2, participants also responded to two measures on purchase convenience: “How convenient is $__ bill for making purchases?”, “How convenient is $__ bill for carrying in a wallet?”; denomination familiarity: “How often do you purchase items using […] bill?” (1 = “never”/10 = “very often”); and product affordability: “How affordable did you find the [product]?” (1 = “not affordable”/10 = “very affordable”). Finally, participants indicated their age, gender, and income levels.

### Results

**FIGURE 4:**
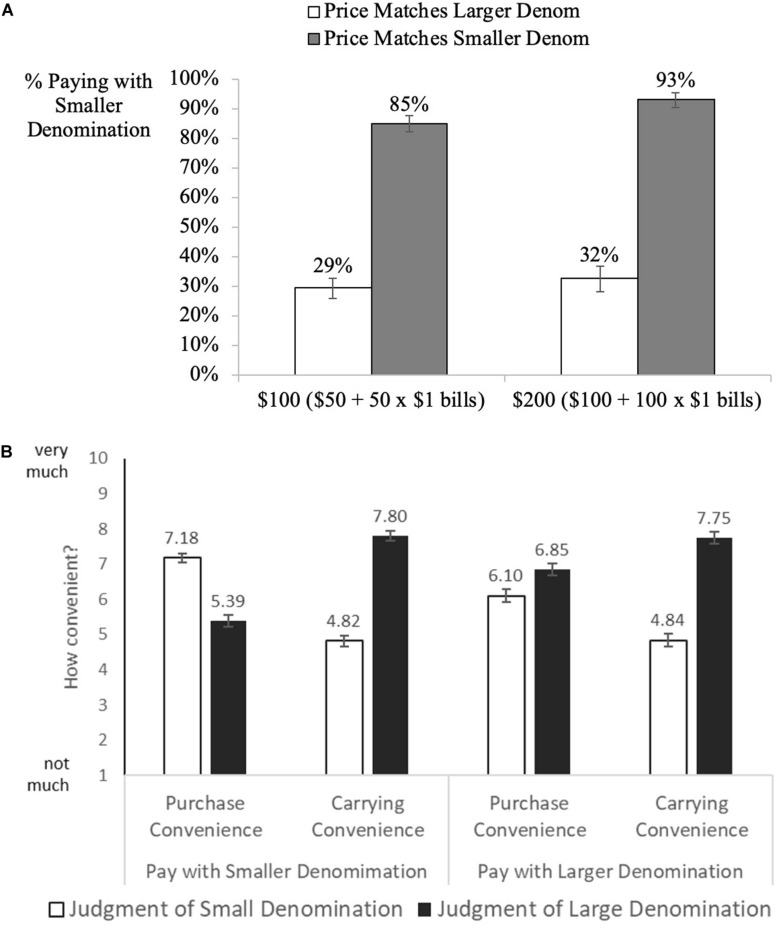
**(A)** Study 4. Influence of the price-denomination effect on denomination choice manipulating storage convenience. Error bars denominate std. errors. **(B)** Study 4. Judgement of purchase and storage convenience by type of bill one paid with. Error bars denominate std. errors.

The dependent variable was the proportion of participants paying with the smaller denomination. As shown in Figure 4A, when participants had to purchase products with prices that matched the larger denomination at hand, they were less likely to use the smaller $1 bills they had ($50 perfume: 29% [χ2(1) = 28.20, *z* = −5.31]; $100 camera: 32% [χ2(1) = 13.37, *z* = −3.66], *p*s < 0.001), and overwhelmingly chose to purchase using the denomination that matched the price of the product. It is worth noting that they could have eliminated their stack of $1 bills that are inconvenient to carry and store; instead, participants chose to hold on to them and pay with the larger denomination, which is evidently easier to carry and store.

On the other hand, when participants had to purchase the $10 shampoo, they were more likely to use their smaller $1 bills compared to using their larger bills ($100 spend level: 85% [χ2(1) = 81.06, *z* = 9.0]; $200 spend level: 93% [χ2(1) = 82.29, *z* = 9.07], *p*s < 0.001). Logistic regressions controlling for the convenience measures as well as other covariates indicate that the effect is robust and that people were less likely to get rid of large number of smaller denominations when the high price anchor was available (log-odds = −2.39,for $50 product, and log-odds = −4.38 for $100 dollar product, *ps* < 0.001, see Supplementary Appendix 6). We do not find any covariates consistently significant for both $100 and $50 product, however some individual covariates were significant for an individual product (see Supplementary Appendix 6).

We further analyze participants’ responses to the convenience measures. First, independent of the condition, all participants (550) judged $1 bills as less convenient to carry than larger bills (*M*_smaller_denom_ = 4.83 vs. *M*_larger_denom_ = 7.78, *t*(549) = 16.44, *p* < 0.001). This indicates that our manipulation worked: driving the number of the smaller denominations to the extreme made people judge smaller denominations as less convenient to carry (compared to carrying convenience in study 3). Regarding purchase convenience, similarly to study 3, on average, participants judged smaller denomination to be more convenient to purchase with than the larger denominations (*M*_smaller_denom_ = 6.75 vs. *M*_larger_denom_ = 5.98, *t*(549) = −4.46, *p* < 0.001). Next, we compared the responses from participants that chose to pay with the larger denominations (*n* = 222) to those who chose to pay with the smaller ones (*n* = 328). Participants in both groups rated larger denominations more convenient to carry (those who chose the smaller denomination: *M*_smaller_denom_ = 4.82 vs. *M*_larger_denom_ = 7.80, *t*(327) = 12.47, *p* < 0.001; those who chose the larger denomination: *M*_smaller_denom_ = 4.84 vs. *M*_larger_denom_ = 7.75, *t*(221) = 10.72, *p* < 0.001), indicating that carrying convenience did not drive their choice of denomination. With regards to purchase convenience, participants who chose the smaller denomination rated it more convenient for making purchases (*M*_smaller_denom_ = 7.18 vs. *M*_larger_denom_ = 5.39, *t*(327) = 8.47, *p* < 0.001) while the opposite was true for those who chose the larger denomination: *M*_smaller_denom_ = 6.10 vs. *M*_larger_denom_ = 6.85, *t*(221) = −2.96, *p* = 0.003), see Figure 4B.

### Discussion

These results lend further support for the price-denomination effect. We replicate the effect even when the number of smaller denominations is stretched to the extreme and when we control for carrying convenience. The findings run contrary to the storage convenience explanation that consumers would get rid of large numbers of smaller denominations. Rather, we find that when the price of the product ($50 perfume or $100 camera) matches the value of the larger denominations, a majority of participants prefer to pay with the larger denomination carried, despite the inconvenience of carrying fifty or one hundred $1 bills.

Studies 2-4 provided initial evidence that “price-denomination effect” can be explained by anchoring, and that the effect is robust beyond storage or purchase convenience (see also Supplementary Tables A2, A3, A12–A15 for storage and purchase convenience results in regressions and results of Supplementary studies). However, one potential explanation for the price-denomination effect is “value-matching” between the subjective value assigned to the product and the denomination. That is, because higher priced products as well as larger denominations might be perceived as more valuable, larger denominations might be used to purchase higher priced products due to value-matching of the perceived product and denomination value. Similarly, people might prefer to buy lower-priced products with smaller denominations because both lower priced-products and smaller denominations have lower “perceived value” in the eyes of the customer. We examine this potential mechanism in study 5. Specifically, in the next study, we investigate whether consumers have a tendency to transfer the value of ownership from the physical money they possess to a product.

## Study 5. Testing Value Matching

Study 5’s aim was to test a potential value matching explanation. Research has shown that people have a sense of higher ownership value for larger bills compared to smaller bills under conditions of increased social presence (Di Muro and Noseworthy, 2013, study 2). Therefore, it is possible that, given such conditions, consumers transfer the value of ownership for money bills they possess to the purchase. This would result in an alternative route that would also predict that consumers will use a lower valued denomination to pay for a lower valued purchase. On the contrary, the price-denomination effect predicts that price information influences choice of which bills consumers choose to purchase with, regardless of perceived product value.

### Method

#### Participants and Design

Four hundred and thirty-eight participants (female = 32%, *M*_*age*_ = 26.75, *SD* = 8.89) from the Prolific Academic panel were recruited in exchange for modest compensation. Participants were assigned at random to either condition in a 2 (purchase price: €10 vs. €50) × 2 (gift type: gift for a jerk boss vs. valentine gift) between-subjects design. The amount of money received including the denominations was held constant across treatment conditions.

#### Materials and Procedure

Participants in the jerk boss’ gift condition imagined that, after receiving a €100 cash bonus (in a single €50 bill and five €10 bills), their work colleagues informed them of their boss’ upcoming anniversary. They all decided to contribute cash (€10 or €50, depending on the assigned condition) towards purchasing a gift for the boss. However, no one, including the participant, liked the boss. Those in the Valentine’s Day gift condition imagined they wanted to purchase a Valentine’s Day gift for a friend. Similar to studies 2 and 4, participants were told how much money they had and the denominations in which they had them. Participants were asked to indicate which denomination they preferred to use in purchasing the gift. This served as our dependent variable. Next, participants responded to a survey that included a measure of purchase convenience based on their response to the denomination choice question: “How convenient was it to pay [€10/€50] for your [boss’/friend’s] gift using a [€10/€50] bill?” (1 = “not at all convenient”/10 = “extremely convenient”), an attention check, gender, age, and income level. Five responses were excluded for failing an attention check, leaving a usable sample of *N* = 433.

### Results

#### Denomination Choice

**FIGURE 5:**
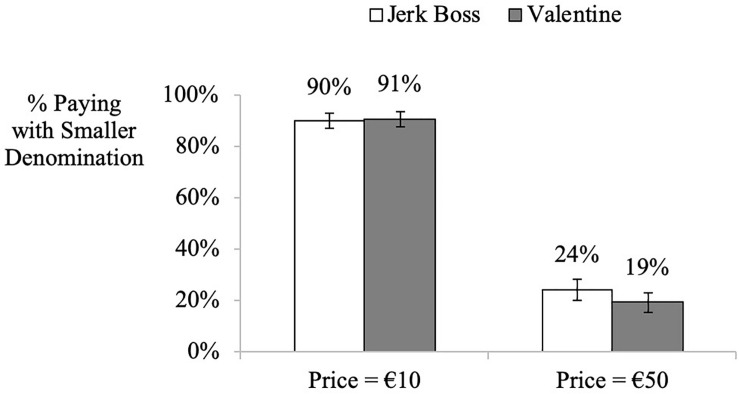
Study 5. Influence of price information on denomination choice manipulating gift type. Error bars denominate std. errors. The *y*-axis represents the proportion of participants who chose to pay with the smaller denomination they possessed. All participants possessed a single €50 bill and five €10 bills.

As shown in Figure 5, participants were more likely to pay using the smaller €10 bills when the price of the purchase matched their smaller bills, regardless of how much they valued the recipient (jerk boss’ gift: 90% [χ2(1) = 70.4, *z* = 8.39]; Valentine gift: 91% [χ2(1) = 70.74, *z* = 8.41], *p*s < 0.001). On the other hand, participants were less likely to use the smaller bills when the donation/purchase matched the larger denomination (jerk boss’ gift: 24% [χ2(1) = 28.27, *z* = −5.32]; Valentine gift: 19% [χ2(1) = 41.18, *z* = −6.42], *p*s < 0.001). See further results on convenience in Supplementary Appendix 6.

### Discussion

Study 5 continues to support and build on previous results. On one hand, we replicate the price-denomination effect under different circumstances. In previous studies 1-4, participants were presented with scenarios where they decided on purchasing items for themselves. We replicate our previous findings in study 5 where participants decided on purchasing a gift for a less valued recipient. Study 5 also eliminates the explanation that greater value being placed on the ownership of larger bills influences our results. Rather, we replicate the price-denomination effect for a low valued recipient but highly priced product. The results indicate that denomination choice is unaffected by value matching tendencies. Instead, participants’ choice of which denomination to purchase the gifts with was determined by the extent to which they relied on price cues.

The results so far suggest that the price-denomination effect can be extended to evaluations of payment mechanisms. It raises the question of whether price cues that trigger paying with denominations will influence payment choice in the presence of a more convenient option – a debit card. Research suggests that if individuals have the option of paying with a card vs cash their willingness-to-pay increases (Prelec and Simester, 2001). Can, therefore, payment with the card (vs. cash) attenuate the price-denomination effect? We suggest that the price-denomination effect would imply that people will use cash when denomination at hand is the same as the price to be paid, while use card when denomination at hand does not match the price. We test this idea in study 6 using an online panel.

## Study 6. Choice of Payment Form

Study 6’s aim is to explore whether individuals are more likely to pay with cash (vs. a debit card) when possessing both payment forms. Previous research suggest that consumers prefer paying with card compared to cash because the former is more convenient (Feinberg, 1986) and associated with lower pain of paying (Prelec and Loewenstein, 1998; Raghubir and Srivastava, 2009). However, the price-denomination effect predicts that individuals will be more likely to pay with cash when the price matches the value of the money bill they hold, but not when it does not match it.

### Method

Participants from Mechanical Turk (*n* = 160; 49% female; *M*_age_ = 40, *SD* = 11.95) were assigned at random to one of four conditions in a 2 (Denominations: two $50 bills vs. ten $10 bills) × 2 (Price: $10 vs. $50) between-subjects design. Participants read a vignette asking them to imagine they traveled by taxi and when they got to their destination, the taxi driver informed them of how much the taxi cost. Participants were told they possessed $100 dollars in either two $50 bills or ten $10 bills, depending on the condition to which they are assigned. Those in the lower price condition were told the taxi ride cost $10 while those in the higher price condition were told the ride cost $50. To control for the decoupling effect (Prelec and Loewenstein, 1998) and cash back benefits (Feinberg, 1986; Soman and Cheema, 2002), participants were further informed that they possessed a debit card, which could easily cover the cost of the ride, and which they could pay with if they so preferred. Next, participants indicated how they would choose to pay for the taxi ride (1 = cash/0 = debit card), as the main dependent measure.

Participants also responded to four convenience measures: “How convenient is it to use a [denomination] to pay for a taxi that costs [price]?”; “How convenient is it to use [debit card] to pay for a taxi that costs [price]?”; “How convenient is it for the taxi driver to receive payment in a [denomination] for a ride that costs [price]?”; “How convenient is it for the taxi driver to receive payment with debit card for a ride that costs [price]?”, all anchored (1 = “not at all convenient”/9 = “very convenient”); and pain of paying: “How much pain are you feeling right now about spending money on the taxi?” (1 = “not painful at all”/9 = “very painful”), adapted from Xu et al. (2015). Finally, participants indicated their age, gender and income levels. Two responses (1% of our sample) were excluded from the analyses due to failed attention check, leaving a usable sample of *N* = 158.

### Results

**FIGURE 6:**
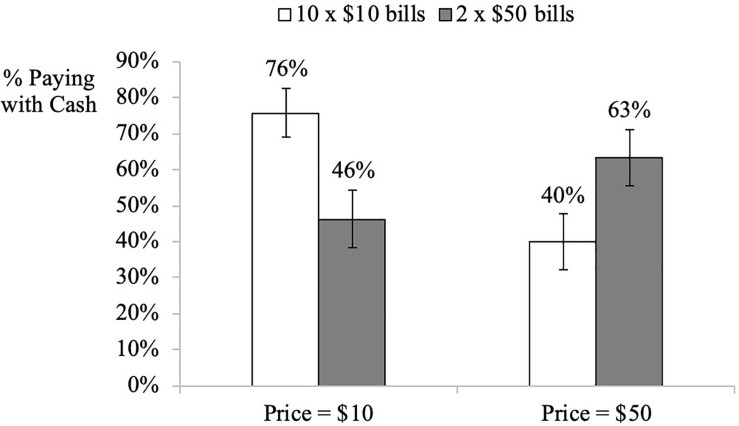
Study 6. Influence of price-denomination effect on payment choice. Error bars denominate std. errors. The *y*-axis represents the proportion of participants who chose to pay with cash.

The dependent variable was the proportion of participants who purchased with cash. As predicted, when the taxi ride cost $10, participants with ten $10 bills (76%) were more likely to pay with cash (vs. debit card) compared to those who had two $50 bills (vs. 46%: χ2(1) = 7.31, *p* = 0.006). In contrast, when the taxi ride cost $50, participants who had two $50 bills (63%) were more likely to pay with cash (vs. debit card) compared to those who had ten $10 bills (40%: χ2(1) = 4.18, *p* = 0.041), see Figure 6.

We also examined whether convenience ratings varied when participants paid with cash (vs. card). One-way ANOVAs revealed that participants who paid with cash (*M* = 7.82, *SD* = 1.69) compared to those who paid with card (*M* = 6.09, *SD* = 2.54) indicated higher convenience for cash payment (*F*(1, 156) = 26.37, *p* < 0.001, η^2^ = 0.17), and higher convenience for others receiving cash payment (*M*_Cash_: 7.56 vs. *M*_Card_: 6.86, *SD*s: 2.07 vs. 2.45, *F*(1, 156) = 3.85, *p* = 0.052, η^2^ = 0.02). Conversely, participants who paid with cash (*M* = 6.85, *SD* = 2.15) compared to those who paid with card (*M* = 8.20, *SD* = 1.51) indicated lower convenience levels for card payment (*F*(1, 156) = 19.69, *p* < 0.001, η^2^ = 0.13), and lower convenience for others to receive card payment (*M*_Cash_: 6.61 vs. *M*_Card_: 7.59, *SD*s: 1.99 vs. 1.86, *F*(1, 156) = 10.13, *p* = 0.002, η^2^ = 0.06). We observed no difference in pain of paying between choice of payment form (*F* < 1). It is unclear whether the convenience ratings reflect the choice that has been made (Cesario et al., 2004), or drive it.

### Discussion

Study 6 results add further evidence of a price-denomination effect. The results suggest that consumers’ payment preference (cash versus card) may shift depending on whether the price they encounter matches the denominations they hold.

## General Discussion

In this paper we investigated the price-denomination hypothesis and found that individuals are likely to choose the denomination that is exactly the same as the price of the product to be purchased (studies 1A-1B). We further examined the role of anchoring on price as a potential mechanism for this effect and found that people make faster decisions when they choose to pay with a denomination that matches the price (study 2). Study 3 showed that the price-denomination effect holds for prices 10% below the denomination value, controlling for storage and purchase convenience and suggested that purchase convenience is unlikely to explain the results. Study 4 replicated the effect even when the number of smaller denominations was stretched to an unusually high number, showing that storage convenience is also unlikely to explain our results. In study 5, we tested whether the value-matching between the product and denomination reduced the price-denomination effect and found no evidence that the effect is driven by value matching. Finally, study 6 tested the extension of the effect to situations in which consumers choose between paying with cash or a debit card. We found that consumers are more likely to use cash over debit card when the price matched the cash denominations the customers held, suggesting that price-denomination match also influences the choice of payment method.

Despite the rise of mobile payments, cash as a payment method is as strong as ever. A majority of consumers pay with cash in most countries (Bagnall et al., 2016; Rosenbaum, 2018). In fact, the amount of cash in circulation has increased over time. In the United States, cash in circulation has grown at ≥ 5% for the past two decades. The number of notes in circulation has doubled to 40 billion between 1996 and 2016. In Europe, the growth is even higher at 6%. It is, therefore, important to understand how consumers handle cash (Matheny et al., 2016; Wheatley, 2017). One of the main features of cash is its denomination. Studying how consumers deal with larger denominations is of particular importance given recent trends in currency circulation – demand for larger denominations rose drastically in the United States after 2008(Matheny et al., 2016; Wheatley, 2017)- as well as recent calls for the elimination of large paper-money (Rogoff, 2016). Does the difference in denominations affect the way we use them? The work we present here suggests that it does, and it is a function of the price of the product one is purchasing. In particular, we find that people pay with denominations that are the same as product price, even when it is more convenient to get rid of smaller denominations. In our studies people consistently showed a tendency to use larger denominations for higher priced products, and lower denominations for lower priced products: the price-denomination effect. This effect replicates across consumers from different continents, online, in the lab and in the field, and using different currencies.

We propose that one of the possible routes through which the effect operates is that a price serves as an anchor that guides the choice of denominations. Results of study 2, which tested anchoring as a possible mechanism as well as measured reaction times, are consistent with this proposed explanation. The theory we present here focuses on specific situations where the price of the product is exactly the same as one of the denominations carried. We also present empirical evidence that adds to the boundary conditions of our findings and shows that this effect goes beyond exact match and holds for prices up to 10% below the denomination. Beyond this deviation, the effect is not stable and attenuates or disappears. How people select denominations in other possible scenarios (for example, when the price is 40% below the larger denomination) is out of scope of this paper but is necessary to study in future research in order to build an all-encompassing theory of the choice of denominations used in different scenarios. We now discuss the implications of this research for theory and practice.

### Theoretical Contributions

Our contribution to previous research is twofold. First, this research contributes to the literature on anchoring on price information. Previous research suggested that consumers use price information as an anchor, and that this anchor influences their information processing, their internal reference price, and ultimately their belief about the value of the product (Morwitz et al., 1998; Thomas and Morwitz, 2005; Chandrashekaran and Grewal, 2006). Our research shows that product price also affects the choice of the denomination consumer pays with. That is, we demonstrate yet another influence that price exercises on consumers as an anchor. Consumers holding small and large denominations choose the small denomination when it is the same as the low price, and the large denominations when it is equal to the high price. Consumers anchor on the price when deciding which denomination to use. In fact, when the product price matches the denomination being held, consumers decide on which denomination to use faster. Our results are aligned with research on judgment anchoring, which also relies on response latencies to examine anchoring effects (Epley and Gilovich, 2001; Mason et al., 2013).

Second, our work contributes to the stream of research on the subjective value of money. Standard economic theory would posit that money is ultimately money, regardless of what we could call circumstantial specificities, such as the method of payment (cash, credit card, etc.), the currency (dollars, euros), or the denomination (ten $10 bills or one $100 bill). But numerous researchers have uncovered violations of this descriptive invariance principle. For instance, when people think about money, they tend to do so in nominal value rather than in real monetary value (Shafir et al., 1997). Relatedly, when people value a product in a foreign currency they have a tendency to be more influenced by the face value, without making the necessary adjustments for the exchange rates (Raghubir and Srivastava, 2002). Also, the salience of money (whether money is introduced earlier or later in a decision) influences how people discount money, which in turn influences their choices and decisions (Jiang et al., 2016). Another way in which consumers violate the descriptive invariance principle is by spending more when they use a credit card versus when they use cash (Raghubir and Srivastava, 2008), and spending more when holding smaller versus larger denominations (Mishra et al., 2006; Raghubir and Srivastava, 2009). Our research is particularly relevant to an area within this body of work: the impact of prices that match denominations (smaller or larger bills) posed on consumer behavior. We investigate whether price influences the choice of denominations, and, accordingly, add to the literature on the subjective value of money.

### Implications

Our work has implications for policymakers as well as businesses. Paper money seems to be at a crossroads. On the one hand, it is not only widely used but actually its circulation increases over time. On the other, there are important (even moral) reasons why certain stakeholders ask for it to being phased out, particularly large bills (Rogoff, 2016). Consider for instance the EU decision in 2016 to discontinue production and issuance of €500 bills, or the Indian Government’s decision in 2016 to demonetize the Rs 500 and Rs 1000 bills and replace them with new Rs 500 and Rs 2000 bills. It is unclear how these moves affect consumer spending in the longer term. Will they lead to lower priced purchases in the EU, and higher priced purchases in India? This research suggests that the denominations in circulation may skew purchases of products and services that match those denominations. Policy makers may, therefore, benefit from a nuanced understanding of how consumers use cash. We hope our research can inform central banks’ decisions on what denominations to issue and keep in circulation. Results from our research could also help commercials banks decide on how to dispense the appropriate denominations from ATMs; a decision that may be contingent on the retail context in which the ATM is located.

Our findings can also be used by managers willing to make their pricing and offerings more persuasive. They could consider adjusting their prices to the amounts represented by the bills in circulation in their particular market. This can be done in different forms: bundling or unbundling, modifying packaging sizes, etc. Such policies might also differ across countries. First, different countries have different denomination values and, therefore, pricing and bundling of products might be influenced by denominations available in the country. Second, one can speculate that stores in those countries where cash is used more often than other payment forms could benefit from such policies more than countries where other electronic methods of payment are dominant. By the same token, consumers would also benefit from understanding the price-denomination effect, and remain vigilant against overspending.

The results also have implications for when people choose between payment forms. Past research suggests that since parting with cash is psychologically more painful than parting with other money forms (Prelec and Loewenstein, 1998; Duclos and Khamitov, 2019), people will spend more using cards as compared to cash (Prelec and Simester, 2001; Soman, 2003; Raghubir and Srivastava, 2008). Our findings from study 6 suggest that, different from what previous work has documented, there is no difference in the pain of parting with money between the cash condition and the card condition. Therefore, the price-denomination effect can possibly be a boundary condition for the “pain of payment” feeling: consumers may choose cash payment over more convenient electronic payment forms when the price is the same as the denomination at hand.

### Areas for Future Research

We believe there are several interesting directions for future research. First, it would be interesting to study the affective consequences of the choice of denominations. The consequences of paying with denominations matching the price of the product may lead to positive emotions. Based on the work by Mishra et al. (2006), it is possible that in situations where consumers have the exact denomination matching the price of the product, they may feel the transaction was more perceptually fluent when they paid using the matching denomination (Alter and Oppenheimer, 2008). This should lead them to feel happier about the transaction and contribute to a positive shopping experience. We suggest this topic as a promising avenue for further research.

In this paper we identified one possible mechanism behind price-denomination effect: price anchoring. Though we found no evidence for some potential explanations of the price-denomination effect (storage convenience, purchase convenience, exact price matching or value being placed on the ownership of larger bills), we could not rule out other alternative explanations. Since most of the effects are multiply-determined, another interesting avenue for further research is to examine the other possible antecedents of price-denomination effect.

What are the possible moderators of price-denomination effect is another topic worth investigating. It would be interesting to explore whether the price denomination effect is weakened/strengthened under condition of time pressure, purchase of healthy vs unhealthy items, or situations where deliberation is encouraged. For example, it is possible that competence in manipulating cash or different abilities in doing mental arithmetic could moderate the price-denomination effect, especially under conditions of cognitive load, or time pressure. For example, imagine that you are running late and need to pay a taxi driver. It is plausible that you would aim to minimize the cognitive effort of doing mental arithmetic, as well as minimize possible errors of the transaction and would be more likely to use the same denominations as the price of the service, not only because of anchoring, but also because of cognitive load and time pressure. The exacerbating effects of time pressure and cognitive load on the price-denomination effect are suggested as future avenues for research.

In the studies reported here, we found that a minority of participants chose to pay for a higher-priced items with smaller denomination even when an exact-matching larger denomination was available, as well as vice versa. This could be a result of noise due to making a random choice in an online study. Consistent with this speculation, the percentage of mismatched denomination choices was the lowest in the field studies 1A and 1B. The minority that did choose to pay with a mismatched bill was always below 50% (likelihood of choosing one of two denominations totally at random, see Table 1). In the studies reported in the Supplementary Appendix 1, however, when the denominations were held in the form of coins (versus notes) a significantly higher proportion of participants than would be indicated by random choice, chose to pay with their smaller denominations. One can speculate that the use of coins may be a boundary condition for the price-denomination effect. While for bills carrying convenience was not at play, coins may be more inconvenient than paper bills, which may affect people’s decisions to choose to pay with them if possible. This research was not designed to compare coins to bills, but it can be an interesting area of future research.

As noted above, our results can also be interpreted using a mental accounting perspective. Mental accounting was primarily conceptualized as a cognitive process “to organize, evaluate, and keep track of financial activities” (Thaler, 1999). Mental accounting has since then been applied to a large number of financial and non-financial behaviors (for a recent review, see Zhang and Sussman, 2018.) While we note this intriguing possibility, we believe future research could formally test whether consumers do, indeed, assign different mental accounts for different denominations: for example, $1’s and $5’s for tips and coffee, $10’s and $20’s for outdoor food, fruit and flower shopping, and $50’s and $100’s for contractor’s services.

One of the limitations of our empirical work is that, for the most part, it assumes that consumers are aware of the bills they carry in their wallet. However, there are systematic biases in the recall of denominations held in one’s wallet that favor the recall of fewer larger denominations over the more numerous smaller ones (Raghubir et al., 2017). This opens up interesting lines of future work. How do consumers react if they realize they do not carry the denomination that matches the price? Do they finalize the purchase? Is their evaluation of the purchase intact? We also did not test how customers feel about their purchases (apart from pain of paying in study 6). Studying the influence of the price-denomination match on satisfaction with purchase is an interesting avenue for further investigation. For example, one could speculate that if a product costs $50 and it turns out that the consumer does not carry this denomination in her wallet and, instead, needs to use smaller bills, she might be less satisfied with the purchase. In addition, prior research has established that consumers have a stronger memory trace of expenses made with cash as compared to credit cards, leading to greater future spending with credit cards as compared to cash (Srivastava and Raghubir, 2002). Therefore, it would be interesting to examine whether the same price and denomination lead to a weaker memory trace than a purchase made when they are different, and consumers engage in cognitively effortful mental arithmetic. Assessing memory and subjective states of the customers facing such purchases are interesting avenues to explore in future research.

Finally, we mainly measured purchase convenience for the purchasing agent directly. Another potential mechanism could be examining the transactional convenience for the person receiving money as a payment (see Supplementary Appendix 7 for some preliminary results for the party receiving cash might matter). For example, Di Muro and Noseworthy (2013) demonstrated that students were more likely to break crisp (vs. worn) bills in a social context, when others could observe their transaction due to pride in ownership of the crisp bill. In a “private” context, however, pride in ownership made participants more likely to purchase with the worn bills. In our context, in study 4, it is possible that people did not want to get rid of fifty $1 bills so as not to be perceived as “cheap” by the experimenter or the party receiving the money.

More generally, it would be interesting to investigate whether the price-denomination effect influences spending decisions, which was out of the scope for our paper. Differences in spending could happen in at least two ways: (*1*) do consumers spend more or less, depending on whether the price of an item matches their denomination at hand?, and (*2*) does carrying specific denominations influence consumer choices of specific products?

We believe these are interesting and relevant questions, and we encourage future research to examine them.

## Data Availability Statement

Data, study protocols and analysis codes are publicly available at https://osf.io/syvrm/. The flows of the field experiments are described in the text directly.

## Ethics Statement

Participants in laboratory and online experimental studies involving human participants provided their written consent to participate in the studies. Participants in the field studies did not provide any written consent due to the nature of the studies (they were not aware of participating in the experiment and rather responded to the promotion of the local retailers with which we partnered for the study). The studies involving human participants were reviewed and approved by Comité de Ética de la Investigación de la Universidad de Navarra.

## Author Contributions

ER, JI, and PR contributed to the idea, design of the studies, data analysis, and results interpretation. JI did the data collection. ER, JI, PR, and IG did the write-up of the manuscript. All authors contributed to the article and approved the submitted version.

## Conflict of Interest

The authors declare that the research was conducted in the absence of any commercial or financial relationships that could be construed as a potential conflict of interest.
