# Supplementary material for: Price-Denomination Effect: Choosing to Pay With Denominations That Are the Same as the Product Prices
Source: Front Psychol. 2020 Sep 9;11:552888. doi: 10.3389/fpsyg.2020.552888 (PMC7509091; doi:10.3389/fpsyg.2020.552888)
Supplement: Supplementary file 1 [file Table_1.DOCX]

Supplementary Material

Table A1. Study descriptions for additional studies reported in Appendices 1, 3 and 4.

|  | Denomination Manipulation | Spend Level | Products | Prices | *N* | Results: |
| --- | --- | --- | --- | --- | --- | --- |
| Appendix 1 | €1 + 5 × €0.20c | €2 | Trident gum | € 1 | 750 | Results^1^:  68% |
|  |  |  | Wrigley gum | €0.20c |  | 97% |
|  | €2 + 4 × €0.50c | €4 | Mints | € 2 |  | 65% |
|  |  |  | Candy | €0.50c |  | 97% |
|  | €20 + 4 × €5 | €40 | Duvet set | € 20 |  | 24% |
|  |  |  | 6 coat hangers | € 5 |  | 92% |
|  | €100 + 5 × €20 | €200 | Jacket | € 100 |  | 12% |
|  |  |  | Headphones | € 20 |  | 91% |
|  | €500 + 5 × €100 | €1,000 | TV | € 500 |  | 11% |
|  |  |  | Compact camera | € 100 |  | 76% |
| Appendix 3 | €50 & 5 $\times$ €10 | €100 | Perfume | €49.99 | 101 | 13% |
|  | €50 & 5 $\times$ €10 |  | Shampoo | €9.99 |  | 92% |
| Appendix 4 | N200 & 4 × N50 | N400 | Notebook | N180 | 99 | 0% |

*Note*.– The dependent variable is the proportion of participants paying with the smaller denomination.

# Appendix 1. Additional Study Showing Robustness of “Price-Denomination Effect” Across Product Categories

## Method

### Participants and Design

Seven hundred and fifty participants across continental Europe (female = 35%, *M*_age_ = 29.67 years, *SD =* 9.50), were recruited from Prolific Academic in exchange for modest compensation. Participants were assigned at random to one of ten conditions in a 2 (purchase price: price matches larger vs. smaller denomination carried) × 5 (spend level, or the amount people imagined they held: €2, €4, €40, €200, and €1,000) between-subjects design. At the lower spend levels (€2 vs. €4) participants handled coins, while at the higher spend levels of €40, €200 and €1,000 participants handled bills. Data was collected in two waves with randomisation and number of participants per condition similar in each wave. We account for wave in our analyses. It did not affect the results.

### Materials and Procedure

Depending on the assigned condition, participants were instructed to imagine they wanted to purchase a product. Participants were asked to further imagine they had only certain amount of money for this purchase (“spend level” mentioned above), and were shown images of the denominations they held. Then, they were asked to imagine they had no other means of payment and could purchase only one unit of that product. Next, participants indicated their choice of denomination to purchase the product by selecting either the larger or the smaller denomination. Finally, participants responded to a survey that elicited responses on purchase and storage convenience for each denomination held. Participants responded to the following questions: “How convenient is [bill] for making purchases?” and “How convenient is [bill] for carrying in a wallet?” (1 = “not at all convenient”/10 = “extremely convenient”); familiarity with the product: “How often do you purchase [product]?” (1 = “never,”/10 = “very often”), adapted from Teixeira et al. (2014); attitude towards the product: “How much do you like [product]?” (1 = “not at all,”/10 = “very much”), adapted from Irmak et al. (2010). Finally, participants reported their age, gender, and income levels.

## Results

The dependent variable was the proportion of participants who choose to purchase with the smaller denomination. The proportions are displayed in figure A1 below.

We start by analysing the condition when the participant held bills (spend level conditions of €40, €200, €1,000). We find that price influences the choice of denomination at hand. When the price was equal to the smaller denomination, 86% of participants chose to purchase with smaller denominations. When the price was equal to the larger denomination, only 16% of participants chose to pay with the smaller denomination. This difference was significant (χ2(1) = 224.79, *p* < 0.001). Logistic regressions controlling for the convenience, familiarity, attitude and the demographic measures, showed that the effects were robust (refer to table below). These results provide further support to H1 and replicate the findings of studies 1A-1B.

In the coins conditions when the price was equal to the smaller denomination, 97% of participants chose to purchase with the smaller denomination as compared to 67% when the price was equal to the larger denomination (χ2(1) = 47.79, *p* < 0.001), although both proportions were higher than 50% implying a preference for smaller denominations at lower price levels.

### Discussion

This study replicates the findings from study 1A-1B for the bill conditions across a wider range of prices and products, using a cross-cultural sample, a different currency, and a different set of denominations. Supporting our main assertion, participants who held bills showed a consistent preference for purchasing with the denomination that reflected the price information encountered. That is, when the price matched the smaller of two denominations, participants preferred to use smaller denominations for the lower priced items. On the other hand, when prices were high, participants preferred to pay with larger denominations.

However, the effect is not without its boundaries. In the smaller spend, coin conditions, though we observe the effect of price on denomination choice, the effect is not as large as for the larger spend level bills. This indicates that the proposed “price-denomination effect” may be stronger for larger (vs. smaller) spend levels as are associated with bills (vs. coins). In the smaller spend, coin conditions, there was an overall preference for the smaller denomination, but this preference was exacerbated when the price matched the smaller denomination carried.

**Supplementary Figure A1.** Proportion paying with smaller denominations when prices matched smaller (vs. larger) denominations*.* The dependent variable is the proportion of participants paying with the smaller denomination. In the money bill conditions, participants relied on price information in choosing which bill to purchase with. In the coin conditions, participants preferred to purchase with smaller denominations regardless of the price level.

Table A2. Logistic regressions for Appendix 1 estimating choice as a function of price anchors and controlling for covariates

| Spend Level | €2 | €4 | €40 | €200 | €1000 |
| --- | --- | --- | --- | --- | --- |
| Intercept | 3.43^*^ | 6.67^**^ | 8.54^***^ | 3.32 | 0.62 |
|  | (1.70) | (2.12) | (2.28) | (1.70) | (1.00) |
| Price Anchor | -3.75^***^ | -3.83^***^ | -4.89^***^ | -4.62^***^ | -3.59^***^ |
|  | (0.91) | (0.93) | (0.79) | (0.65) | (0.57) |
| Convenience: Purchasing with Larger Denomination | 0.14 | -0.06 | -0.23 | -0.15 | -0.22 |
|  | (0.15) | (0.15) | (0.15) | (0.14) | (0.12) |
| Convenience: Purchasing with Smaller Denomination | 0.20 | -0.01 | -0.31^*^ | 0.09 | 0.34^*^ |
|  | (0.12) | (0.12) | (0.15) | (0.16) | (0.14) |
| Convenience: Carrying Larger Denomination | 0.10 | 0.06 | 0.25 | -0.02 | 0.03 |
|  | (0.14) | (0.13) | (0.15) | (0.11) | (0.11) |
| Convenience: Carrying Smaller Denomination | -0.41^**^ | -0.25 | -0.27^*^ | 0.06 | -0.07 |
|  | (0.13) | (0.13) | (0.13) | (0.15) | (0.12) |
| Product Familiarity | -0.10 | -0.13 | 0.10 | 0.15 | -0.07 |
|  | (0.16) | (0.16) | (0.17) | (0.20) | (0.17) |
| Product Attitude | 0.07 | -0.00 | 0.04 | -0.16 | 0.01 |
|  | (0.16) | (0.16) | (0.14) | (0.14) | (0.10) |
| Female | -0.26 | -0.01 | -0.20 | 0.11 | -0.05 |
|  | (0.57) | (0.53) | (0.57) | (0.64) | (0.50) |
| Age | -0.00 | 0.02 | -0.05 | -0.03 | 0.00 |
|  | (0.03) | (0.03) | (0.03) | (0.03) | (0.02) |
| Wave 1 | 0.51 | 1.44^*^ | 0.49 | -0.50 | 1.01^*^ |
|  | (0.55) | (0.56) | (0.52) | (0.59) | (0.49) |
| AIC | 118.78 | 122.51 | 128.13 | 117.34 | 141.07 |
| BIC | 151.97 | 155.56 | 161.18 | 150.53 | 174.19 |
| Log Likelihood | -48.39 | -50.26 | -53.07 | -47.67 | -59.54 |
| Deviance | 96.78 | 100.51 | 106.13 | 95.34 | 119.07 |
| Num. obs. | 151 | 149 | 149 | 151 | 150 |
| ^***^*p* < 0.001, ^**^*p* < 0.01, ^*^*p* < 0.05 | |  |  |  |  |

*Notes*.– Standard errors are in parenthesis. Dependent variable (1 = pay using smaller denomination; 0 otherwise). Participants were less likely to purchase with smaller denominations when prices matched larger bills in hand. The dummy variable “wave” (1 = “wave 1”, 0 = “wave 2”) indicates that part of the data was collected within a one-month difference, due to budget constraints. The dummy variable “Price Anchor” indicates treatment conditions where price matched the larger denomination, coded as follows:

€2 Spend Level (1 = €1 Trident, 0 = 0.25¢ Wrigley)

€4 Spend Level (1 = €2 Mints, 0 = 0.50¢ Candy)

€40 Spend Level (1 = €20 Duvet, 0 = €5 Hangers)

€200 Spend Level (1 = €100 Jacket, 0 = €20 Headphones)

€1000 Spend Level (1 = €500 TV, 0 = €100 Camera)

# Appendix 2

In the main text we reported the analysis for log-transformed latencies. Here we report non-log transformed actual latencies in seconds. A one-way ANOVA on reaction time as a function of price information including all covariates, revealed a main effect of price information (*F*(2, 1137) = 5.29, *p* = 0.005, *η*^2^ = 0.01), product attitude (*F*(1, 1137) = 6.88, *p* = 0.009, *η*^2^ = 0.01), age (*F*(1, 1137) = 31.88, *p* < 0.001, *η*^2^ = 0.03), and gender (*F*(1, 1137) = 7.18, *p* = 0.007, *η*^2^ = 0.01), indicating that the speed at which participants responded to the product varied depending on whether or not prices matched denominations held. The effect of matching was similar when the covariates were excluded from the model (*F*(2, 1142) = 5.15, *p* = 0.006, *η*^2^ = 0.01).

We further present t-test for non-logged reaction times. On average, participants who faced a price that was an exact match for the denomination they carried responded faster (*M* = 8.49) compared to those who saw the $30 price (*M* = 10.19, *t*(763) = -3.38, *p* = 0.001, Cohen’s *d* = 0.24), or those who saw no price (*M* = 10.23, *t*(762) = -2.70, *p* = 0.007, Cohen’s *d* = 0.20). Results from linear regression models controlling for covariate measures indicate that these results are robust.

Table A3 Logistic regression for study 2 (shampoo and perfume products)

Denomination choice as a function of price anchors controlling for covariates

|  | Model 1 | Model 2 |
| --- | --- | --- |
| Intercept | 0.31^**^ | 0.26 |
|  | (0.10) | (0.29) |
| Price = Equidistant Anchor | -0.97^***^ | -1.23^***^ |
|  | (0.15) | (0.22) |
| Price = Exact Match Anchor | -0.14 | 1.46^***^ |
|  | (0.15) | (0.27) |
| Shampoo |  | -0.56^*^ |
|  |  | (0.24) |
| Shampoo * Equidistant Anchor |  | 0.52 |
|  |  | (0.30) |
| Shampoo * Exact Match Anchor |  | -3.05*** |
|  |  | (0.36) |
| Attitude towards Product |  | 0.00 |
|  |  | (0.00) |
| Familiarity with Product |  | 0.00 |
|  |  | (0.00) |
| Age |  | 0.01 |
|  |  | (0.01) |
| Female |  | -0.13 |
|  |  | (0.14) |
| Income |  | 0.01 |
|  |  | (0.02) |
| AIC | 1542.65 | 1335.31 |
| BIC | 1557.78 | 1390.79 |
| Log Likelihood | -768.32 | -656.66 |
| Deviance | 1536.65 | 1313.31 |
| Num. obs. | 1145 | 1145 |
| ^***^*p* < 0.001, ^**^*p* < 0.01, ^*^*p* < 0.05 | | |
|  | | |

*Notes.*– Standard errors are in parenthesis. Dependent variable (1 = purchase with larger denomination). The dummy variable “Price Anchor: Exact Match” indicates treatment conditions where price matched the denomination carried (1 = subject faced ExactMatch condition; 0 = otherwise). The dummy variable “Price Anchor: Equidistant Match” indicates subject faced treatment conditions where price=30 (1 = subject faced Equidistant Match condition; 0 = otherwise). Shampoo is the dummy variable indicating product (shampoo = 1 if the product is shampoo; 0= if the product is perfume). Attitude and familiarity were measured on 100-point Likert-type scales. Higher scores indicate higher levels on the continuous variable. The results indicate that participants in the “price = exact match” condition were more likely to purchase using the larger denominations they had compared to those in the “no price” condition.

Table A4. Logistic regression for study 2 (perfume product)

Denomination choice as a function of price anchors controlling for covariates

|  | Model 1 | Model 2 |
| --- | --- | --- |
| Intercept | 0.59^***^ | -0.09 |
|  | (0.15) | (0.40) |
| Price = Equidistant Anchor | -1.24^***^ | -1.23^***^ |
|  | (0.21) | (0.22) |
| Price = Exact Match Anchor | 1.44^***^ | 1.50^***^ |
|  | (0.27) | (0.27) |
| Attitude towards Product |  | 0.00 |
|  |  | (0.00) |
| Familiarity with Product |  | -0.00 |
|  |  | (0.00) |
| Age |  | 0.02^*^ |
|  |  | (0.01) |
| Female |  | -0.32 |
|  |  | (0.21) |
| Income |  | 0.00 |
|  |  | (0.03) |
| AIC | 644.03 | 647.19 |
| BIC | 657.12 | 682.10 |
| Log Likelihood | -319.01 | -315.59 |
| Deviance | 638.03 | 631.19 |
| Num. obs. | 581 | 581 |
| ^***^*p* < 0.001, ^**^*p* < 0.01, ^*^*p* < 0.05 | | |

*Notes*.– Standard errors are in parenthesis. The regression equation above only applies to treatment conditions where participants encountered the perfume product. Dependent variable (1 = purchase with larger denomination). The dummy variable “Price Anchor: Exact Match” indicates treatment conditions where price matched the denomination carried (1 = subject faced ExactMatch condition; 0 = otherwise). The dummy variable “Price Anchor: Equidistant Match” indicates subject faced treatment conditions where price=30 (1 = subject faced Equidistant Match condition; 0 = otherwise ). Attitude and familiarity were measured on 100-point Likert-type scales. Higher scores indicate higher levels on the continuous variable. The results indicate that participants in the “price = exact match” condition were more likely to purchase using the larger denominations they had compared to those in the “no price” condition.

Table A5. Logistic regression for study 2 (shampoo product)

Denomination choice as a function of price anchors controlling for covariates

|  | Model 1 | Model 2 |
| --- | --- | --- |
| Intercept | 0.03 | -0.05 |
|  | (0.15) | (0.45) |
| Price = Equidistant Anchor | -0.69^**^ | -0.70^***^ |
|  | (0.21) | (0.21) |
| Price = Exact Match Anchor | -1.57^***^ | -1.56^***^ |
|  | (0.24) | (0.24) |
| Attitude towards Product |  | -0.00 |
|  |  | (0.00) |
| Familiarity with Product |  | 0.00 |
|  |  | (0.00) |
| Age |  | -0.00 |
|  |  | (0.01) |
| Female |  | 0.04 |
|  |  | (0.19) |
| Income |  | 0.01 |
|  |  | (0.03) |
| AIC | 684.04 | 693.71 |
| BIC | 697.04 | 728.39 |
| Log Likelihood | -339.02 | -338.85 |
| Deviance | 678.04 | 677.71 |
| Num. obs. | 564 | 564 |
| ^***^*p* < 0.001, ^**^*p* < 0.01, ^*^*p* < 0.05 | | |

*Notes*.– Standard errors are in parenthesis. The regression equation above only applies to treatment conditions where participants encountered the shampoo product. Dependent variable (1 = purchase with larger denomination). The dummy variable “Price Anchor: Exact Match” indicates treatment conditions where price matched the denomination carried (1 = subject faced ExactMatch condition; 0 = otherwise). The dummy variable “Price Anchor: Equidistant Match” indicates subject faced treatment conditions where price=30 (1 = subject faced Equidistant Match condition; 0 = otherwise. The dummy variable “Price Anchor: Exact Match” indicates treatment conditions where price matched the denomination carried (1 = subject faced ExactMatch condition; 0 = otherwise). The dummy variable “Price Anchor: Equidistant Match” indicates subject faced treatment conditions where price=30 (1 = subject faced Equidistant Match condition; 0 = otherwise ). Shampoo is the dummy variable indicating product (shampoo=1 if the product is shampoo; 0= if the product is perfume). Attitude and familiarity were measured on 100-point Likert-type scales. Higher scores indicate higher levels . The results indicate that participants in the “price = exact match” condition were less likely to purchase using the larger denominations they had (or more likely to purchase using their smaller bills) compared to those in the “no price” condition.

Table A6. OLS regressions for Study 2 estimating log_10_ response latencies response latencies as a function of price anchors and relevant price information, controlling for covariates

|  | Model 1 | Model 2 |
| --- | --- | --- |
| Intercept | 2.10^***^ | 1.54^***^ |
|  | (0.03) | (0.08) |
| Price = Equidistant Anchor | 0.02 | -0.04 |
|  | (0.05) | (0.06) |
| Price = Exact Match Anchor | -0.15^**^ | -0.30^***^ |
|  | (0.05) | (0.06) |
| Shampoo |  | 0.07 |
|  |  | (0.07) |
| Shampoo * Equidistant Anchor |  | 0.14 |
|  |  | (0.09) |
| Shampoo * Exact Match Anchor |  | 0.34^***^ |
|  |  | (0.09) |
| Attitude towards Product |  | 0.00^*^ |
|  |  | (0.00) |
| Familiarity with Product |  | 0.00 |
|  |  | (0.00) |
| Age |  | 0.01^***^ |
|  |  | (0.00) |
| Female |  | -0.07 |
|  |  | (0.04) |
| Income |  | -0.01^**^ |
|  |  | (0.01) |
| R^2^ | 0.01 | 0.14 |
| Adj. R^2^ | 0.01 | 0.13 |
| Num. obs. | 1145 | 1145 |
| RMSE | 0.63 | 0.59 |
| ^***^*p* < 0.001, ^**^*p* < 0.01, ^*^*p* < 0.05 | | |

*Notes.–* Standard errors are in parenthesis. The dependent measure was the log_10_ time (in seconds) it took participants to choose which denomination to purchase with, given price information encountered. The dummy variable “Price Anchor: Exact Match” indicates treatment conditions where price matched the denomination carried (1 = subject faced ExactMatch condition; 0 = otherwise). The dummy variable “Price Anchor: Equidistant Match” indicates subject faced treatment conditions where price=30 (1 = subject faced Equidistant Match condition; 0 = otherwise ). Shampoo is the dummy variable indicating product (shampoo=1 if the product is shampoo; 0= if the product is perfume). Attitude and familiarity were measured on 100-point Likert-type scales. Higher scores indicate higher levels Attitude and familiarity were measured on 100-point Likert-type scales. Higher scores indicate higher levels Participants responded faster when they purchased with a denomination that matched the price compared to when there was no price information.

Table A7. OLS regressions for Study 2 estimating log_10_ response latencies (in seconds) as a function of price anchors and relevant price information, controlling for covariates (shampoo only)

|  | Model 1 | Model 2 |
| --- | --- | --- |
| Intercept | 2.08^***^ | 1.55^***^ |
|  | (0.05) | (0.13) |
| Price = Equidistant Anchor | 0.10 | 0.10 |
|  | (0.07) | (0.06) |
| Price = Exact Match Anchor | 0.06 | 0.04 |
|  | (0.07) | (0.06) |
| Attitude towards Product |  | 0.00 |
|  |  | (0.00) |
| Familiarity with Product |  | 0.00 |
|  |  | (0.00) |
| Age |  | 0.01^***^ |
|  |  | (0.00) |
| Female |  | -0.13^*^ |
|  |  | (0.05) |
| Income |  | -0.01 |
|  |  | (0.01) |
| R^2^ | 0.00 | 0.09 |
| Adj. R^2^ | 0.00 | 0.07 |
| Num. obs. | 564 | 564 |
| RMSE | 0.65 | 0.62 |
| ^***^*p* < 0.001, ^**^*p* < 0.01, ^*^*p* < 0.05 | | |

*Notes.–* Standard errors are in parenthesis. The regression equation above only applies to treatment conditions where participants encountered the shampoo product. The dependent measure was the log_10_ time (in seconds) it took participants to choose which denomination to purchase with, given price information encountered. The dummy variable “Price Anchor: Exact Match” indicates treatment conditions where price matched the denomination carried (1 = subject faced ExactMatch condition; 0 = otherwise). The dummy variable “Price Anchor: Equidistant Match” indicates subject faced treatment conditions where price=30 (1 = subject faced Equidistant Match condition; 0 = otherwise). Attitude and familiarity were measured on 100-point Likert-type scales. Higher scores indicate higher levels Attitude and familiarity were measured on 100-point Likert-type scales. Higher scores indicate higher levels Participants responded faster when they purchased with a denomination that matched the price compared to when there was no price information.

Table A8. OLS regressions for Study 2 estimating log_10_ response latencies (in seconds) as a function of price anchors and relevant price information, controlling for covariates (perfume only)

|  | Model 1 | Model 2 |
| --- | --- | --- |
| Intercept | 2.11^***^ | 1.50^***^ |
|  | (0.04) | (0.10) |
| Price = Equidistant Anchor | -0.07 | -0.04 |
|  | (0.06) | (0.06) |
| Price = Exact Match Anchor | -0.34^***^ | -0.30^***^ |
|  | (0.06) | (0.06) |
| Attitude towards Product |  | 0.00 |
|  |  | (0.00) |
| Familiarity with Product |  | 0.00 |
|  |  | (0.00) |
| Age |  | 0.02^***^ |
|  |  | (0.00) |
| Female |  | -0.02 |
|  |  | (0.05) |
| Income |  | -0.02^*^ |
|  |  | (0.01) |
| R^2^ | 0.06 | 0.18 |
| Adj. R^2^ | 0.05 | 0.17 |
| Num. obs. | 581 | 581 |
| RMSE | 0.59 | 0.56 |
| ^***^*p* < 0.001, ^**^*p* < 0.01, ^*^*p* < 0.05 | | |

*Notes.*– Standard errors are in parenthesis. The regression equation above only applies to treatment conditions where participants encountered the perfume product. The dependent measure was the log_10_ time (in seconds) it took participants to choose which denomination to purchase with, given price information encountered. The dummy variable “Price Anchor: Exact Match” indicates treatment conditions where price matched the denomination carried (1 = subject faced ExactMatch condition; 0 = otherwise). The dummy variable “Price Anchor: Equidistant Match” indicates subject faced treatment conditions where price=30 (1 = subject faced Equidistant Match condition; 0 = otherwise). Attitude and familiarity were measured on 100-point Likert-type scales. Higher scores indicate higher levels Attitude and familiarity were measured on 100-point Likert-type scales. Higher scores indicate higher levels Participants responded faster when they purchased with a denomination that matched the price compared to when there was no price information.

Table A9. OLS regressions for Study 2 estimating response latencies (in seconds, not logged) as a function of price anchors and relevant price information, controlling for covariates

|  | Model 1 | Model 2 |
| --- | --- | --- |
| Intercept | 10.23^***^ | 4.73^***^ |
|  | (0.44) | (1.10) |
| Price = Equidistant Anchor | -0.05 | -0.32 |
|  | (0.62) | (0.85) |
| Price = Exact Match Anchor | -1.74^**^ | -2.53^**^ |
|  | (0.62) | (0.85) |
| Shampoo |  | 0.49 |
|  |  | (0.94) |
| Shampoo * Equidistant Anchor |  | 0.68 |
|  |  | (1.21) |
| Shampoo * Exact Match Anchor |  | 1.74 |
|  |  | (1.21) |
| Attitude towards Product |  | 0.03^**^ |
|  |  | (0.01) |
| Familiarity with Product |  | 0.00 |
|  |  | (0.01) |
| Age |  | 0.11^***^ |
|  |  | (0.02) |
| Female |  | -1.22^*^ |
|  |  | (0.52) |
| Income |  | -0.08 |
|  |  | (0.08) |
| R^2^ | 0.01 | 0.06 |
| Adj. R^2^ | 0.01 | 0.06 |
| Num. obs. | 1145 | 1145 |
| RMSE | 8.56 | 8.35 |
| ^***^*p* < 0.001, ^**^*p* < 0.01, ^*^*p* < 0.05 | | |

*Notes.–* Standard errors are in parenthesis. The dependent measure was the time (in seconds) it took participants to choose which denomination to purchase with, given price information encountered. The dummy variable “Price Anchor: Exact Match” indicates treatment conditions where price matched the denomination carried (1 = subject faced ExactMatch condition; 0 = otherwise). The dummy variable “Price Anchor: Equidistant Match” indicates subject faced treatment conditions where price=30 (1 = subject faced Equidistant Match condition; 0 = otherwise). Shampoo is the dummy variable indicating product (shampoo=1 if the product is shampoo; 0= if the product is perfume). Attitude and familiarity were measured on 100-point Likert-type scales. Higher scores indicate higher levels Participants responded faster when they purchased with a denomination that matched the price compared to when there was no price information.

Table A10. OLS regressions for Study 2 estimating response latencies (in seconds, not logged) as a function of price anchors and relevant price information, controlling for covariates (shampoo only)

|  | Model 1 | Model 2 |
| --- | --- | --- |
| Intercept | 10.61^***^ | 6.96^**^ |
|  | (0.76) | (2.11) |
| Price = Equidistant Anchor | 0.41 | 0.29 |
|  | (1.06) | (1.06) |
| Price = Exact Match Anchor | -0.57 | -0.80 |
|  | (1.07) | (1.06) |
| Attitude towards Product |  | 0.05^*^ |
|  |  | (0.02) |
| Familiarity with Product |  | -0.02 |
|  |  | (0.02) |
| Age |  | 0.08^*^ |
|  |  | (0.03) |
| Female |  | -2.13^*^ |
|  |  | (0.90) |
| Income |  | 0.07 |
|  |  | (0.14) |
| R^2^ | 0.00 | 0.03 |
| Adj. R^2^ | -0.00 | 0.02 |
| Num. obs. | 564 | 564 |
| RMSE | 10.33 | 10.23 |
| ^***^*p* < 0.001, ^**^*p* < 0.01, ^*^*p* < 0.05 | | |

*Notes.*– Standard errors are in parenthesis. The regression equation above only applies to treatment conditions where participants encountered the shampoo product. The dependent measure was the time (in seconds) it took participants to choose which denomination to purchase with, given price information encountered. The dummy variable “Price Anchor: Exact Match” indicates treatment conditions where price matched the denomination carried (1 = subject faced ExactMatch condition; 0 = otherwise). The dummy variable “Price Anchor: Equidistant Match” indicates subject faced treatment conditions where price=30 (1 = subject faced Equidistant Match condition; 0 = otherwise). Attitude and familiarity were measured on 100-point Likert-type scales. Higher scores indicate higher levels Participants responded faster when they purchased with a denomination that matched the price compared to when there was no price information.

Table A11. OLS regressions for Study 2 estimating response latencies (in seconds, not logged) as a function of price anchors and relevant price information, controlling for covariates (perfume only)

|  | Model 1 | Model 2 |
| --- | --- | --- |
| Intercept | 9.87^***^ | 4.13^***^ |
|  | (0.45) | (1.03) |
| Price = Equidistant Anchor | -0.52 | -0.26 |
|  | (0.64) | (0.61) |
| Price = Exact Match Anchor | -2.83^***^ | -2.47^***^ |
|  | (0.63) | (0.60) |
| Attitude towards Product |  | 0.02 |
|  |  | (0.01) |
| Familiarity with Product |  | 0.01 |
|  |  | (0.01) |
| Age |  | 0.14^***^ |
|  |  | (0.02) |
| Female |  | -0.64 |
|  |  | (0.54) |
| Income |  | -0.21^**^ |
|  |  | (0.08) |
| R^2^ | 0.04 | 0.15 |
| Adj. R^2^ | 0.03 | 0.13 |
| Num. obs. | 581 | 581 |
| RMSE | 6.25 | 5.92 |
| ^***^*p* < 0.001, ^**^*p* < 0.01, ^*^*p* < 0.05 | | |

*Notes*.– Standard errors are in parenthesis. The regression equation above only applies to treatment conditions where participants encountered the perfume product. The dependent measure was the time (in seconds) it took participants to choose which denomination to purchase with, given price information encountered. The dummy variable “Price Anchor: Exact Match” indicates treatment conditions where price matched the denomination carried (1 = subject faced ExactMatch condition; 0 = otherwise). The dummy variable “Price Anchor: Equidistant Match” indicates subject faced treatment conditions where price=30 (1 = subject faced Equidistant Match condition; 0 = otherwise). Attitude and familiarity were measured on 100-point Likert-type scales. Higher scores indicate higher levels Participants responded faster when they purchased with a denomination that matched the price compared to when there was no price information.

# Appendix 3

Table A12. Logistic regression for Study 3: Denomination choice as a function of price anchors controlling for covariates, for camera

|  | Model 1 |
| --- | --- |
| Intercept | 2.03^*^ |
|  | (0.88) |
| Price = $80 | 0.12 |
|  | (0.29) |
| Price = $90 | 1.57^***^ |
|  | (0.34) |
| Price = $100 | 0.70^*^ |
|  | (0.30) |
| Convenience of Purchasing with Larger Bill | -0.02 |
|  | (0.05) |
| Convenience of Carrying Larger Bill | -0.05 |
|  | (0.04) |
| Convenience of Purchasing with Smaller Bill | -0.23^*^ |
|  | (0.09) |
| Convenience of Carrying Smaller Bill | 0.11^*^ |
|  | (0.06) |
| Familiarity with Larger Bill | -0.07 |
|  | (0.07) |
| Familiarity with Smaller Bill | 0.04 |
|  | (0.05) |
| Familiarity with Camera | -0.18^*^ |
|  | (0.09) |
| Attitude towards Camera | 0.12^*^ |
|  | (0.05) |
| Affordability of Camera | -0.04 |
|  | (0.05) |
| Female | -0.59^**^ |
|  | (0.23) |
| Age | 0.01 |
|  | (0.01) |
| Income | -0.06 |
|  | (0.03) |
| AIC | 539.22 |
| BIC | 604.71 |
| Log Likelihood | -253.61 |
| Deviance | 507.22 |
| Num. obs. | 443 |
| ^***^*p* < 0.001, ^**^*p* < 0.01, ^*^*p* < 0.05 | |

*Notes.–* The regression equation above only applies to treatment conditions where participants encountered camera products. The dependent variable is coded (1 = purchase with larger denomination). Compared to participants who decided on purchasing the $70 camera, those in the $90 camera and $100 camera treatment conditions were more likely to purchase using the larger bills they had.

Table A13. Logistic regression for Study 3: Denomination choice as a function of price anchors controlling for covariates, for perfume

|  | Model 1 |
| --- | --- |
| Intercept | 0.42 |
|  | (0.75) |
| Price = $40 | 0.12 |
|  | (0.29) |
| Price = $45 | 1.98^***^ |
|  | (0.37) |
| Price = $50 | 1.97^***^ |
|  | (0.37) |
| Convenience of Purchasing with Larger Bill | 0.05 |
|  | (0.05) |
| Convenience of Carrying Larger Bill | -0.09 |
|  | (0.05) |
| Convenience of Purchasing with Smaller Bill | 0.03 |
|  | (0.07) |
| Convenience of Carrying Smaller Bill | -0.02 |
|  | (0.06) |
| Familiarity with Larger Bill | 0.12 |
|  | (0.07) |
| Familiarity with Smaller Bill | -0.04 |
|  | (0.05) |
| Familiarity with Perfume | -0.11 |
|  | (0.07) |
| Attitude towards Perfume | 0.06 |
|  | (0.06) |
| Affordability of Perfume | -0.02 |
|  | (0.05) |
| Female | -0.15 |
|  | (0.24) |
| Age | -0.01 |
|  | (0.01) |
| Income | 0.04 |
|  | (0.04) |
| AIC | 485.03 |
| BIC | 550.35 |
| Log Likelihood | -226.52 |
| Deviance | 453.03 |
| Num. obs. | 438 |
| ^***^*p* < 0.001, ^**^*p* < 0.01, ^*^*p* < 0.05 | |

*Notes.–* The regression equation above only applies to treatment conditions where participants encountered perfume products. The dependent variable is coded (1 = purchase with larger denomination). Compared to participants who decided on purchasing the $35 perfume, those in the $45 perfume and $50 perfume treatment conditions were more likely to purchase using the larger bills they had.

# Appendix 4. Additional Study Showing Robustness of “price-denomination effect” with Prices that are not a Perfect Match to denominations (Prices Ending in .99)

Mason et al. (2013) show that in negotiation contexts, offers presented as precise prices (e.g., $19 or $21) are thought of as more credible than offers presented as rounded prices (e.g., $20). Moreover, in every-day situations, rounded prices are relatively rare in comparison to precise prices ending in .99 for instance. Therefore, this study aimed to test whether our results hold in more realistic price contexts, when prices do not exactly match the denomination at hand but are close to the denomination value. We expect to observe the “price-denomination effect” regardless of whether prices are rounded or precise.

## Method

### Participants and Design

One hundred and one MBA students (female = 29%, *M*_age_ = 29.22, *SD =* 2.47) from a European Business School, agreed to take part in a lab experiment. Participants were assigned at random to one of two conditions in a two-cell between-subjects design that manipulated price at two levels (€49.99 perfume vs. €9.99 shampoo).

### Materials and Procedure

Participants were told they had a hypothetical spending budget of €100 (a €50 bill and five €10 bills). However, in comparison to previous studies, they encountered a product which was priced either at €49.99 (perfume) or €9.99 (shampoo). Next, participants responded to measures on transactional and storage convenience, and product familiarity (see table A14). Finally, they indicated their age, gender, and income level. This design enabled us to robustly test whether the price-denomination hypothesis still holds when people encounter more realistic, precise prices.

## Results

The dependent variable was the proportion of participants paying with smaller denominations. Overall, the proportion of participants paying with the smaller denomination between price conditions differed (Fisher’s exact test, *p* = 0.029). As predicted, when participants had to purchase the €9.99 shampoo they were more likely to use the smaller denominations (€9.99 shampoo: 92%, χ2(1) = 31.69, z = 5.77, *p* < 0.001). However, when participants had to purchase the €49.99 perfume, they became less likely to use the smaller bills (€49.99 perfume: 87%, χ2(1) = 27.25, z = 5.36, *p* < 0.001). Logistic regressions controlling for the covariate measures indicate that the effect is robust (see below).

### Discussion

The results from this study add to the evidence that the “price-denomination effect” holds with precise prices when they are close to denomination values.

Table A14. Logistic regression for Appendix 3 estimating choice as a function of price anchors and controlling for covariates

|  | Larger Denomination  (€50 bill) |
| --- | --- |
| Intercept | 2.91 |
|  | (4.88) |
| Price Anchor: €50 bill | -5.45^***^ |
|  | (1.01) |
| How convenient is €50 for making purchases? | 0.14 |
|  | (0.14) |
| How convenient is €10 for making purchases? | -0.39 |
|  | (0.23) |
| How convenient is €50 for carrying in a wallet? | 0.21 |
|  | (0.14) |
| How convenient is €10 for carrying in a wallet? | -0.01 |
|  | (0.18) |
| How often do you purchase [product]? | 0.28 |
|  | (0.19) |
| Female | 1.56 |
|  | (0.88) |
| Age | -0.04 |
|  | (0.15) |
| AIC | 75.25 |
| BIC | 98.60 |
| Log Likelihood | -28.62 |
| Deviance | 57.25 |
| Num. obs. | 99 |
| ^***^*p* < 0.001, ^**^*p* < 0.01, ^*^*p* < 0.05 |  |

*Notes*.– Standard errors are in parenthesis. Dependent variable (1 = pay using smaller denomination; 0 otherwise). The dummy variable “Price Anchor” indicates treatment conditions where price matched the larger denomination carried (1 = €50 bill; 0 = 5 × €10 bills). In the Model, two responses were dropped due to incomplete data for the age variable. The scale for the convenience questions: was 1 = “not at all convenient”/10 = “extremely convenient” and for product familiarity: 1 = “never,”/10 = “very often” (Teixeira et al., 2014).

# Appendix 5. Additional Field Study Showing Robustness of “Price-Denomination Effect” with a Price Lower than the Denomination Value

## Method

### Participants and Design

Ninety-nine students (female = 54%) who were responding to an on-campus sales promotion in Africa participated in the study. All participants saw one product, which was priced 10% below the larger denomination they had.

### Materials and Procedure

Similar to studies 1A-1B, each participant received a bonus of N400 (≈ U.S $1.1 or €0.97) in a single N200 bill and four N50 bills. The money was contained in a sealed brown envelope and was theirs to keep on the condition that they purchased only one unit of the item on sale–notebook priced at a N180. After paying for the item, participants were given the notebook, issued a receipt, and exited the scene. A duplicate copy of each receipt was used to enter participants’ data for analyses.

## Results

Two responses were excluded due to incomplete data. The dependent variable was the proportion of participants paying with the smaller denomination. The results show that no participant (0%) chose to purchase the notebook using the smaller N50 bills they had. Thus, the results lend further support to the price-denomination hypothesis.

### Discussion

Based on the above results, we can conclude that pure matching is unlikely to explain “price-denomination effect”. In more natural purchase settings where prices differ from denominations up to 20%, denomination decisions were based on the price encountered. Consumers used the denomination that was closest to the price even when the price did not match it.

# Appendix 6

Table A15. Logistic regressions for Study 4 estimating choice as a function of price anchors controlling for covariates

|  | Larger Denomination | |
| --- | --- | --- |
|  | $50 bill | $100 bill |
|  | Model 1 | Model 2 |
| Intercept | -0.40 | 2.94^*^ |
|  | (0.93) | (1.47) |
| Price Anchor | -2.39^***^ | -4.38^***^ |
|  | (0.39) | (0.85) |
| How convenient is $100 for making purchases? | -0.03 | -0.08 |
|  | (0.06) | (0.08) |
| How convenient is $50 for making purchases? | 0.19^**^ | 0.15 |
|  | (0.07) | (0.09) |
| How convenient is $100 for carrying in a wallet? | 0.07 | 0.11 |
|  | (0.07) | (0.09) |
| How convenient is $50 for carrying in a wallet? | -0.10 | -0.12 |
|  | (0.06) | (0.08) |
| How often do you purchase items using $100 bill? | 0.01 | 0.02 |
|  | (0.08) | (0.10) |
| How often do you purchase items using $50 bill? | -0.03 | -0.08 |
|  | (0.06) | (0.08) |
| How often do you purchase [product]? | 0.14 | -0.18 |
|  | (0.09) | (0.14) |
| How much do you like [product] | -0.06 | 0.08 |
|  | (0.07) | (0.10) |
| How affordable did you find the [product]? | 0.02 | 0.11 |
|  | (0.07) | (0.09) |
| Female | 0.96^**^ | 0.07 |
|  | (0.31) | (0.41) |
| Age | 0.01 | -0.01 |
|  | (0.01) | (0.02) |
| AIC | 340.27 | 201.04 |
| BIC | 389.66 | 245.15 |
| Log Likelihood | -157.14 | -87.52 |
| Deviance | 314.27 | 175.04 |
| Num. obs. | 330 | 220 |
| ^***^*p* < 0.001, ^**^*p* < 0.01, ^*^*p* < 0.05 |  |  |

*Notes*.– Standard errors are in parenthesis. Dependent variable (1 = pay using smaller denomination; 0 otherwise). The dummy variable “Price Anchor” indicates treatment conditions where price information matched the larger denomination carried. All scales were 1-10: convenience: 1 = “not at all convenient”/10 = “extremely convenient”; familiarity: 1 = “never,”/10 = “very often”; product attitude: 1 = “not at all,”/10 = “very much”, and product affordability: 1 = “not affordable”/10 = “very affordable”.

# Appendix 7. Additional results study 5.

In addition to testing choice of denomination, we tested whether purchase convenience differed between participants whose denomination choice mirrored the donation/ purchase price versus those whose choice did not. In comparison to convenience measures in other studies, in study 5 we collected responses only on purchase convenience and each subject evaluated only the bills/he had used. Note, than in other studies participants evaluated both — smaller and larger — bills they had at their disposal. Participants who purchased with a denomination matching the price indicated higher purchase convenience levels than their counterparts, who purchased with a denomination not matching the price (*F*(1, 431) = 75.19; *M*_DM_ = 8.84 vs. *M*_noDM_ = 6.66, *SD*s = 1.76 vs. 2.56, *t*(431) = 8.67, , *p*s < 0.001, Cohen’s *d* = 1.15). A two-sided Mann-Whitney test conducted to account for the unbalanced nature of the cells (DC = 365 vs. no-DC = 68), as well as unequal variances between cells indicated that the results were consistent with the *t*-test (*W* = 19436, 95% CI[1.49, 2.35], *p* < 0.001).

# Appendix 8

We have used Rosenthal (1978) meta-analysis method of testing mean *p*-values to verify whether our results hold on globally, that is, across all the studies. We used the following formula for calculating z-value: z = (0.5- mean p)/(sqrt (12*N)), where “mean p” is the mean of *p*-values from all studies that used exact match of prices and bills ( 9 studies in total), and N is the total number of p values which tested exact match hypothesis (26 p values in total). For each study there is usually more than one *p-*value that can be included in the analysis, and we used all *p*-values that applied. For example, for field study 1A, we used both 1) *p*-value testing whether participants used larger denomination in a proportion that is significantly different from chance (50%) when the price was high and matched this denomination, and 2) p-value testing whether participants used smaller denomination in a proportion that is significantly different from chance (50%) when price was matching smaller denomination. We always used *p*-values from two-sided test. For study 2 which tested anchoring, we used only *p*-values for exact-matching anchor; for study 3 we used *p*-values only for exact-matching denominations. For the study in Appendix1, we used only bill conditions since bill conditions were the main focus of this paper. We do not use the study from Appendix 4 for meta-analysis as there was no condition with exact match in this study. We also note, that our calculations are conservative. Most p-values are *ps* <0.001 (e.g., *p* = 0.0003), however, for our calculations we use the p-values as they are reported in this paper, i.e., rounded to 3 digits, and instead of exact small numbers we use *p* = 0.001. For exact p-values used per study, please, refer to table A16.

The “price-denomination” effect is significant: z = 8.78; *p* < 0.001, if we use N=26 (number of p- values used for analysis); or z = 5.17; *p* < 0.001, if we use N = 9 (number of studies used for analysis).

Table A16. P-values used for meta-analysis

| p-values used for Z calculation^1^ | Conditions used | Study | Comments |
| --- | --- | --- | --- |
| 0.001 | high price=large denomination | 1A |  |
| 0.001 | low price= small denomination |  |  |
| 0.001 | high price=large denomination | 1B |  |
| 0.001 | low price= small denomination |  |  |
| 0.001 | high price=large denomination | 2 | condition only for exact match anchor |
| 0.001 | low price= small denomination |  |  |
| 0.001 | for P=100$ | 3 | only for exact matching (prices= 100$ or 50$;do not use non-matching conditions (prices 90$, 80$,70$ or 45$, 40$ or 35$) |
| 0.001 | for P=50$ |  |  |
| 0.001 | P=100$, using 100$ to pay |  |  |
| 0.001 | P=50$, using $50 to pay | 4 |  |
| 0.001 | P=10$, using 1$, endowment 100$ |  |  |
| 0.001 | P=10$, using 1$, endowment 200$ |  |  |
| 0.001 | using 50$ for gift priced at P= 50$ for jerk boss |  |  |
| 0.001 | using 50$ for Valentine´s gift P=50$ | 5 |  |
| 0.001 | using 10$ for gift priced at P=10$ for jerk boss |  |  |
| 0.001 | using 10$ for valentine´s gift priced at P=10$ |  |  |
| 0.041 | using 50$ cash for p=50$ instead of Debit card | 6 |  |
| 0.006 | using 10$ cash for p=10$ purchase instead of debt card |  |  |
| 0.001 | using 20 euro bill for duvet priced at P=20 euro |  | bills only, no coins conditions |
| 0.001 | using 5 euro bill for hangers priced at P=5 euro |  |  |
| 0.001 | using 100 euro bill for buying jacket priced at P=100 euro | Appendix1 |  |
| 0.001 | using 20 euro for buying headphones priced at P=20 euro |  |  |
| 0.001 | using 500 euro bill for buying TV priced at P=500 euro |  |  |
| 0.001 | using 100 euro for buying camera priced at P=100 euro |  |  |
| 0.001 | using 10$ bill for buying shampoo priced at P= 9.99$ | Appendix3 |  |
| 0.001 | using 50$ for perfume priced at 49.99$ |  |  |

^1:^ We use p=0.001 for all p<0.001. This assumption is conservative given we are using larger p-values than the actual p-values obtained in these comparisons

# References

Irmak, C., Vallen, B., and Sen, S. (2010). You like what I like, but I don’t like what you like: uniqueness motivations in product preferences. *J. Consum. Res.* 37, 443–455. doi:10.1086/653139.

Mason, M. F., Lee, A. J., Wiley, E. A., and Ames, D. R. (2013). Precise offers are potent anchors: Conciliatory counteroffers and attributions of knowledge in negotiations. *J. Exp. Soc. Psychol*. 49, 759–763. doi:10.1016/j.jesp.2013.02.012.

Rosenthal, R. (1978). Combining Results of Independent Studies. *Psychol. Bull*. 85, 185–193. doi:10.1037/0033-2909.85.1.185

Teixeira, T., Picard, R., el Kaliouby, R. (2014). Why, When, and How Much to Entertain Consumers in Advertisements? A Web-Based Facial Tracking Field Study. *Mark. Sci.* 33, 763–884. [doi:10.1287/mksc.2014.0854](https://doi.org/10.1287/mksc.2014.0854)
